# Effects of ambient temperature on electric vehicle electricity consumption are reduced in a warming world

**DOI:** 10.1016/j.isci.2025.113328

**Published:** 2025-08-27

**Authors:** Qingyang Wu, Yifang Zhu

**Affiliations:** 1 Department of Environmental Health Sciences, Jonathan and Karin Fielding School of Public Health, University of California, Los Angeles, Los Angeles, CA 90095, USA; 2 Institute of Environment and Sustainability, University of California, Los Angeles, Los Angeles, CA 90095, USA

**Keywords:** Climatology, Energy engineering, Energy systems, Energy management

## Abstract

Emerging evidence suggests that ambient temperature affects electric vehicles (EVs) electricity consumption through influencing lithium-ion batteries, yet this effect remains unclear in a warming world. We simulate global EV stock and electricity consumption patterns in response to temperature changes from 2010 to 2100 on 0.5° grids. Global EV electricity consumption reached 61 TWh in 2022, projected to rise to 679 (654–713) TWh by 2030 and 2,395 (2,151–2,673) TWh by 2050. While overall electricity demand grows with EV adoption, temperature’s percentage impact on this demand decreases from 12.3% in 2030 to 10.0% by 2100. As global warming progresses, temperature’s impact on EV power demand is expected to decline in most nations. However, equatorial areas and Southern Hemisphere mid-latitudes may experience increased consumption. This apparent paradox occurs because massive EV growth outweighs efficiency gains from reduced temperature effects. These findings underscore the need for climate mitigation strategies and energy system planning.

## Highlights

• EV electricity consumption shows a U-shaped response to ambient temperature
• Global EV electricity demand to reach 679 TWh by 2030 and 2,395 TWh by 2050
• Temperature’s relative impact on EV demand declines from 12.3% to 10.0% by 2100
• Despite global trends, some equatorial regions will see increased EV energy demand

## Graphical abstract

**Figure:**
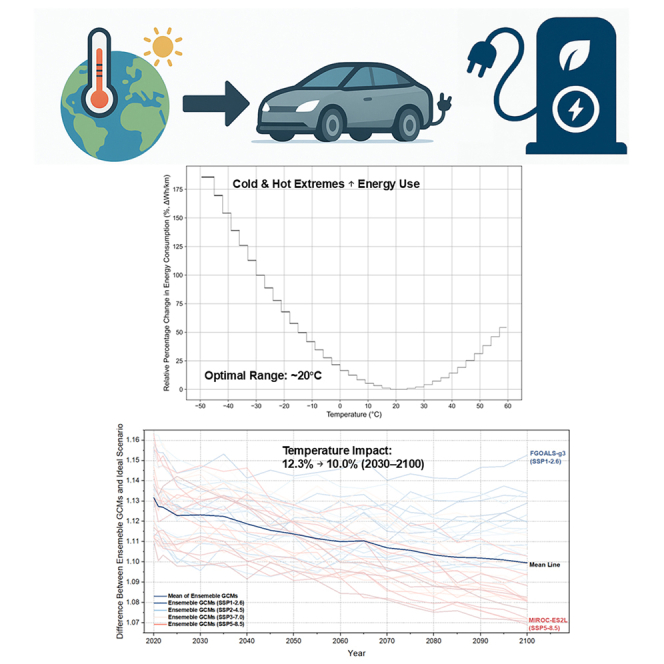

## Introduction

The greenhouse gas (GHG) emissions due to fossil fuel combustion has pushed the global climate and weather systems toward tipping points.^1^ Roughly one-fourth of global GHG emissions are from the transportation sector, of which about 70% are from on road vehicles.^2^ Adopting electric vehicles (EVs) represents a critical measure for countries to fulfill their Nationally Determined Contributions and attain net-zero emissions.^3^^,^^4^ Nevertheless, as EVs are increasingly integrated into broader and more extreme weather contexts, recent observations have uncovered a direct correlation between ambient temperature and electricity consumption of EVs.^5^^,^^6^^,^^7^ One of the key anticipated benefits from EVs is the reduction of GHG emissions; however, if climate change-induced temperature fluctuations alter the demand for electricity, the expected benefits need adjustment.^2^^,^^8^^,^^9^ When EVs replace internal combustion engine vehicles, they shift energy demand from petroleum to electricity. This affects total energy consumption—typically reducing it through higher powertrain efficiency of electric motors compared to combustion engines, though this advantage varies substantially under temperature extremes. The net climate benefits depend on electricity generation sources and operational conditions.

Existing research indicates that lithium-ion batteries, the power source for EVs, are sensitive to ambient temperatures.^7^^,^^10^^,^^11^^,^^12^ Temperature fluctuations affect battery internal resistance and cycle life, subsequently impacting the EVs’ range. Low temperatures impede electrolyte flow and ion transfer, reducing battery capacity and power output, thus shortening driving range.^5^^,^^13^^,^^14^ Studies conducted in the United Kingdom and the United States demonstrate that range may decrease by up to 30% in moderately cold regions and by as much as 50% in colder locales.^10^^,^^15^ While cold weather poses challenges, hot environments also increase electricity demand due to more intensive cooling system use, which can lead to reduced driving range. In hot regions such as Kuwait, the regular use of air conditioning to sustain driver and passenger comfort exacerbates electricity consumption, increasing range anxiety for owners.^16^^,^^17^ Even in climates traditionally deemed favorable, such as California, evidence suggests that global warming contributes to battery overheating, degradation, and premature aging, resulting in traffic disruptions in certain areas.^18^

The global EV market is experiencing exponential growth, with over 26 million EVs in operation worldwide in 2022, marking a nearly 60% surge from 2021. China, Europe, and the United States are major markets.^19^ European countries like Norway are leaders in EV adoption rates.^19^ Electricity consumption for EV operation is projected to account for 3.5% of total electricity demand globally by 2030, with 5.9% in the United States, 5.2% in Europe, and 3.9% in China.^19^ In 2022, rapid growth in EV sales was also witnessed in emerging markets and developing economies (EMDEs), such as India, Thailand, and Indonesia.^19^ These countries are generally relied on grid electricity to power EVs where the electricity is primarily from fossil fuels combustion.^20^ Since EVs produce no tailpipe carbon dioxide equivalent (CO_2_e) emissions, but do cause indirect emissions from power plants, widespread adoption could lead to an increase in CO_2_e emissions.^2^^,^^9^^,^^21^^,^^22^

Our research builds upon recent studies that assessed the interaction between electricity consumption of EVs and ambient temperature within controlled laboratory settings.^14^^,^^23^^,^^24^ While certain studies have focused on specific cities,^5^^,^^17^^,^^18^ individual country,^10^^,^^13^^,^^16^^,^^25^ or experimental fleets,^17^^,^^26^ few encompassing extensive geographical areas or adopting a global perspective or future projection. This limitation is due to several challenges. First, the optimal operational temperatures for EVs and their electricity consumption in response to ambient temperature fluctuations need to be characterized across different areas. Second, projecting additional electricity demand for EVs necessitates simulating future EV stocks across these regions while accounting for long-term and dynamic trends in ambient temperature induced by climate change. Finally, the specific impact of changing ambient temperature by future EV fleets in a warming world on energy demands requires further evaluation.

**Figure 1:**
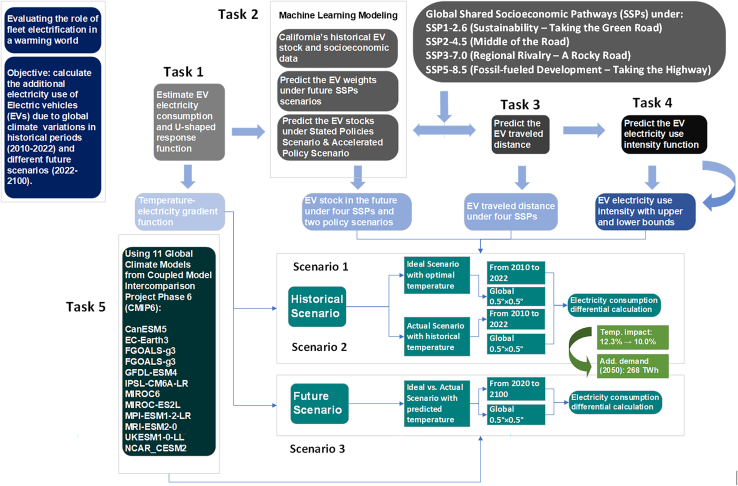
Integrated methodological framework for evaluating the impact of ambient temperature on EV electricity consumption The framework consists of five interconnected tasks. Task 1 establishes the temperature-dependent electricity consumption response function for electric vehicles (EVs) through a meta-analysis of real-world fleet data. Task 2 forecasts future EV stock distribution by downscaling national projections to 0.5° grids using a machine learning model. Task 3 estimates annual vehicle-miles traveled (VMT) per EV based on functions dependent on gross domestic product (GDP). Task 4 models improvements in EV technical efficiency following an exponential learning curve. Finally, Task 5 integrates these components with monthly temperatures from 11 general circulation models (GCMs) under four Shared Socioeconomic Pathways (SSPs) to simulate electricity demand from 2010 to 2100 for both an “actual scenario” (with temperature effects) and an “ideal scenario” (without temperature effects).

To tackle these challenges, we developed an integrated approach, blending modeling and empirical methods. First, we computed the response function of EV electricity usage to ambient temperature across global 0.5 × 0.5° grids. Then, we estimated battery electric vehicle (BEV) and plug-in hybrid electric vehicle (PHEV) stocks on each grid from 2010 to 2100. Finally, we estimated the effect of ambient temperature on EV electricity consumption from additional power demand. Our integrated methodological framework is illustrated in Figure 1.This research reveals that climate change has dual effects on EV electricity consumption. (1) At the global level, warming reduces the percentage impact of ambient temperature on electricity consumption from 12.3% in 2030 to 10.0% by 2100. (2) Despite this reduced relative sensitivity, the absolute electricity consumption continues to rise substantially due to massive EV deployment. Additionally, regional variations exist, with equatorial and Southern Hemisphere regions potentially experiencing increased temperature-related consumption due to higher cooling demands. Our research findings provide policymakers with insights into the effects of climate change on EV electricity consumption, which might inform future sustainable transportation and energy management policies.

## Results

### EV electricity consumption affected by ambient temperature

**Figure 2:**
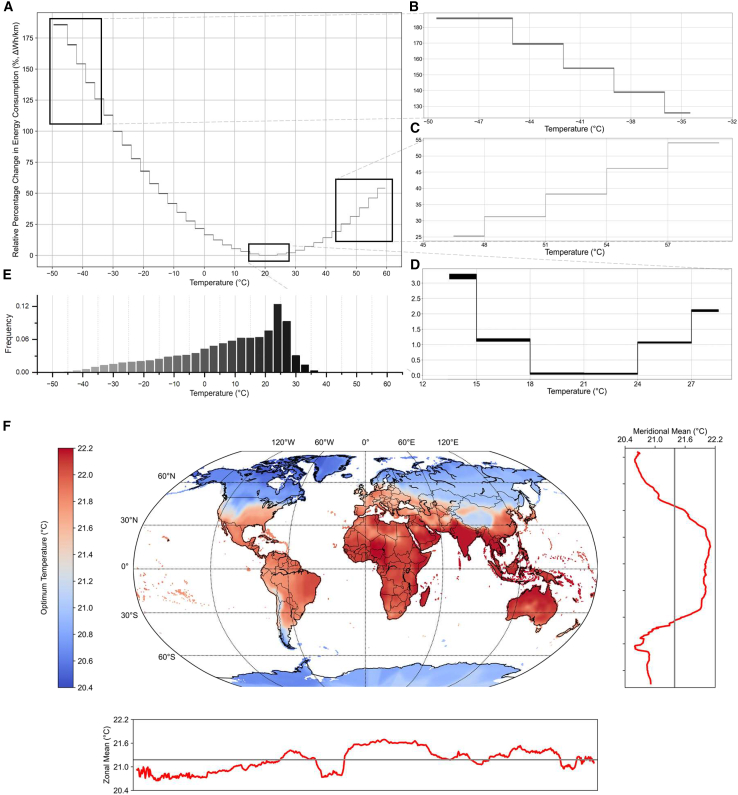
The relationship between EV electricity consumption and global ambient temperature (A–D) Response functions of EV electricity consumption to monthly average temperatures estimated for all 0.5 × 0.5° grids. (A) EV electricity consumption’s U-shaped response to ambient temperature. The temperature range is divided into 36 intervals with a 3 °C interval, from temperatures below −48 °C to those above 57 °C. On the right side, magnified views are provided from top to bottom for (B) cold temperature intervals (from −50 °C to −32 °C), (C) hot temperature intervals (from 45 °C to 60 °C), and (D) the suitable temperature interval (from 15 °C to 27 °C). (E) A histogram of global historical monthly average temperatures from 2010 to 2022. (F) The spatial distribution of the optimal temperatures for EVs in different regions worldwide. Colors range from blue to red, indicating temperatures from low to high. Black contours represent national borders. The zonal and meridional mean of these temperatures are shown on the bottom and right sides of the world map, respectively.

As shown in Figure 2A, EV electricity consumption exhibits a U-shaped response to ambient temperature. Electricity consumption tends to remain low within moderate temperature ranges of 18°C–24 °C (Figures 2A and 1D). Deviations from this optimal range lead to a substantial increase in electricity consumption. Extreme cold environments impact EV electricity consumption (Figure 2B), whereas temperature rises also result in increased energy usage, albeit to a lesser extent compared to colder climates (Figure 2C). Figure 2E shows global historical monthly average temperatures primarily range from −10°C to 30 °C, with occurrences of extreme cold but lower frequencies of extreme heat. This pattern indicates that EVs often operate under ambient temperatures outside the optimal range, leading to increased electricity consumption. Figure 2F illustrates the global spatial variations in the optimal temperature for EVs that exhibit greater variations along latitudes than along longitudes. Higher optimal temperatures are observed in tropical or subtropical regions, including areas of Africa, the Middle East, South Asia, Southeast Asia, and South America. Conversely, average optimal temperatures in Canada, Russia, Northern Europe, and Central Asia generally lie below the global mean. These regions frequently undergo distinct seasonal changes, enduring harsh and frigid winters. As climate change intensifies, ambient temperatures across various regions are changing.^27^ The increased frequency and severity of extreme temperature events, both high and low, can disrupt current patterns of electricity consumption by EVs, thereby imposing additional burden on power generating systems.

### Future global EV stock at the grid level

**Figure 3:**
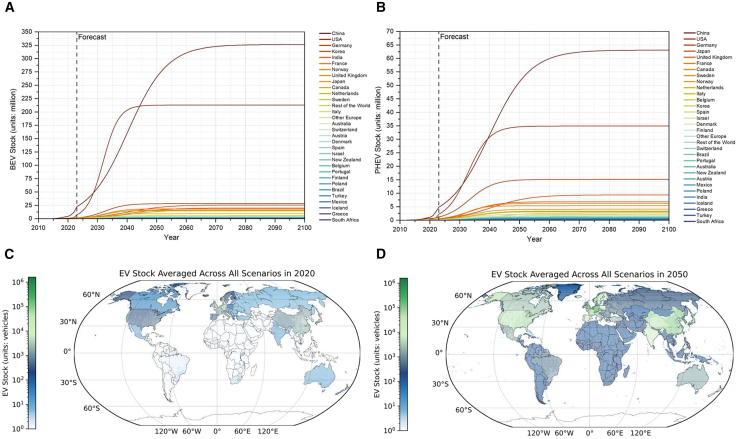
Global EV stock forecast and grid-level downscaling (A–D) The stock of (A) BEVs and (B) PHEVs in various countries during 2010–2100. Gray vertical lines divide the historical and forecast periods, and the color bar on the right indicates different countries. Countries are ordered according to the average stock of EVs throughout the entire period. The total number of EVs stocks across countries for (C) 2020 and (D) 2050 is downscaled into 0.5° × 0.5° grids.

Utilizing multisource data fusion and feature-selected random forest models, we forecast the dynamic evolution of EVs at the national level for future scenarios (see STAR Methods). Figures 3A and 3B present BEV and PHEV stock across 33 major countries and regions from 2010 to 2100. The total EV stock is projected to increase by over 8 times, from 28 million in 2022 to 230 million in 2030, with an annual growth rate of 30%. This forecast closely aligns with the International Energy Agency (IEA)’s Global EV Outlook for 2023 and 2024. The two reports forecast 240 million and 236 million EVs by 2030, respectively.^28^^,^^29^ For individual countries, most will experience steady growth in EV stock, albeit at varying rates. China, the United States, Western Europe, and East Asian countries maintain high EV stock levels throughout the forecast period. EMDEs, particularly India, Israel, and Brazil, are projected to undergo faster EV growth rates. By the mid-to-late century, under the STEPS (Stated Policies Scenario), there will be 830 million EVs on the road by 2050, increasing to 922 million by 2100. Under APS (Alternative Policies Scenario), by 2030, the global EV stock is expected to reach 236 million, 2.8% higher than the STEPS scenario (230 million) and in line with the IEA projection of 236 million.^29^ Under APS, there will be 857 million EVs on the road by 2050, with annual growth rates of 6.7% (Tables S1–S5).

Downscaling the EV stock from the national level to the 0.5° × 0.5° grid level requires identifying the factors influencing EVs distribution within each grid. To this end, we developed a machine learning model based on data in California to establish the predictive framework. As a leader in the United States and worldwide, California has the largest fleet and sales volume of EVs, with comprehensive, accurate, and diverse statistics provided by government agencies.^30^^,^^31^ In addition, California’s Mediterranean climate, mild winters, and dry summers provide ideal conditions for EV operation.^18^ Based on various data collected from California between 2015 and 2019, feature engineering revealed that population size and its interactions with gross domestic product (GDP) are the most important determinants for fleet electrification (see Figure S1A). Trained on these California data, the machine learning framework demonstrated excellent generalization performance across 9,060 census tracts in California in 2020 (see Figures S1B and S1C). Utilizing global gridded GDP and population data, we downscaled the EV stock data for each country to the grid level (approximately 55 km × 55 km near the equator). Since the average daily driving distance for most EVs is often less than 55 km,^32^^,^^33^ this size is sufficient to capture the impact of ambient temperature on EV electricity consumption under consistent climatic conditions.^34^^,^^35^

As shown in Figures 3C and 3D, global EV stock is estimated to rapidly grow over the coming decades. In 2020, EV stock was highest in North America and northwestern Europe. By 2050, the number of EVs is expected to increase dramatically, especially in densely populated mid-latitude regions of the Northern Hemisphere. Europe will see balanced growth in EV stock, with notable increases in Poland, Czechia, and Spain. The adoption of EVs in China will spread nationwide, covering both suburban and rural areas. EV penetration will also continue to rise in Japan and South Korea. South American countries like Brazil and Argentina, African nations such as South Africa, and Australia may also witness large-scale EV use.

### Historical EV electricity consumption

**Figure 4:**
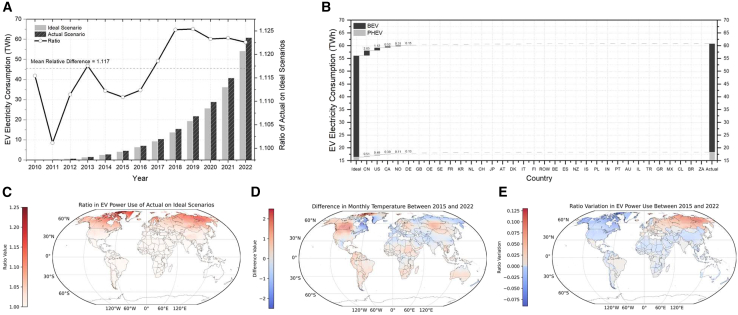
The historical relationship between EV electricity consumption and ambient temperature (A) Between 2010 and 2022, the actual scenario (black bars) and ideal scenario (gray bars) of EV electricity consumption. The line graph shows the ratio between the actual scenario and the ideal scenario, with a horizontal dashed line representing the average level during the historical period. (B) The difference for EV electricity consumption between the actual scenario and the ideal scenario in 2022 is decomposed across countries, with abbreviations following the ISO 3166-1 alpha-2 standard (refer to Table S7). Black and gray bars represent BEV and PHEV, respectively. (C) The ratio of EV electricity consumption between the actual and ideal scenarios in 2022. (D and E) (D) Changes in the global monthly average temperature between 2015 and 2022 and (E) associated changes in the ratio of EV electricity consumption. The actual scenario here accounts for ambient temperature, while the ideal scenario excludes it.

Electricity consumption of EVs depends on the number of stocks, vehicle-miles traveled (VMT), electricity efficiency, and ambient temperature. Figure 4A illustrates the trends in EV electricity consumption from 2010 to 2022, comparing ideal scenarios (i.e., without considering ambient temperature impacts) with actual scenarios where temperature effects are accounted for. In 2022, the electricity consumption of EVs was 54 TWh under ideal scenarios, compared to 61 TWh in actual scenarios. The average ratio between the two is 11.7% over the historical period. Figure 4B and Table S6 illustrate that in 2022, for BEVs, the ideal electricity consumption was 38 TWh, with an actual value differing by 4.7 TWh. The largest contribution to this gap came from China (2.1 TWh), followed by the United States (1.1 TWh) and Canada (0.5 TWh). The impact of temperature fluctuations on PHEVs was minor, with China contributing 0.5 TWh, followed by the United States (0.5 TWh), Canada (0.3 TWh), and Germany (0.1 TWh).

Temperature factors historically increased EV energy burdens across regions (Figure 4C). Near the Arctic Circle, such as in northern Canada and Siberia, actual consumption far exceeded ideals levels. In contrast, in warm climates like Central America and equatorial Africa, consumption was lower than in high latitudes, though extreme heat could require the use of air conditioning. Temperate regions with cold winters and seasonal heating/cooling swings like Central/Eastern Europe, eastern China, and the eastern United States coast generally have suitable temperatures for EV driving.

Historical ambient temperature changes (Figure 4D) have already affected EVs’ electricity consumption (Figure 4E). High-latitude regions in the Northern Hemisphere, such as Northern and Northwestern Canada, Northern United States, and parts of the North Atlantic, have experienced substantial warming, leading to a decrease in EV electricity consumption. Similarly, regions like Scandinavia, Eastern Europe, Southeast Asia, and Northeast China have also warmed, albeit to a lesser extent, which correlates with reduced EV energy usage. These observations are mirrored in the Southern Hemisphere, though to a lesser degree. Certain equatorial regions, such as parts of Central America, Eastern Canada, the Mediterranean, and Northern Australia, have undergone slight cooling. These temperature changes have resulted in increased EV electricity consumption in these areas. Most regions exhibit a consistent trend where rising temperatures lead to reduced power use by EVs. Nevertheless, inconsistencies exist. For instance, historical warming trends in southern and mid-latitude regions of South America have been associated with a slight increase in EV electricity consumption. This can be attributed to the historical climatic suitability of the local environment, where moderate temperature increases can accelerate battery aging.^18^ In tropical regions, even minor temperature rises can potentially increase air conditioning demand.

### Future EV electricity consumption under different climate scenarios

**Figure 5:**
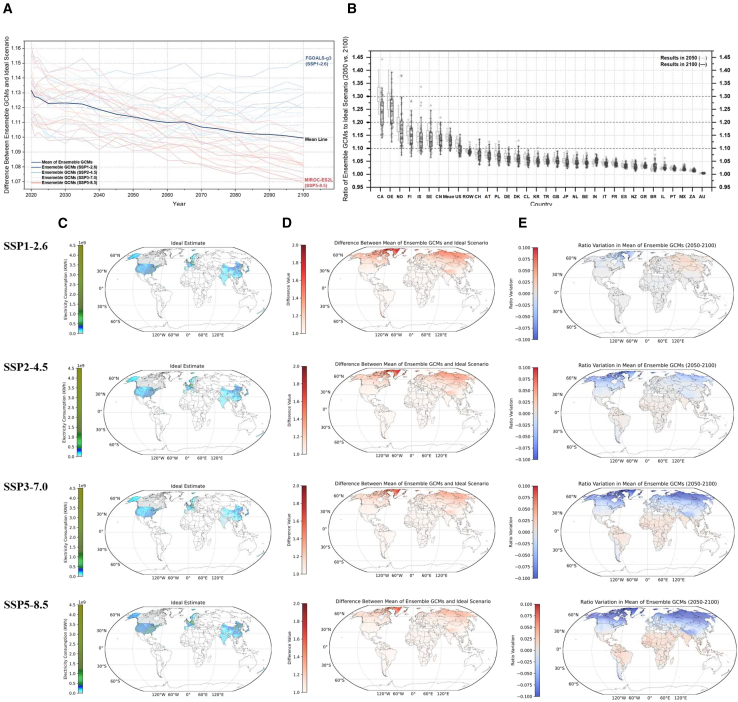
EV electricity consumption attributed to ambient temperature is reduced under global warming (A) Ensemble general circulation models (GCMs) show the trend of EV electricity demand driven by ambient temperature under four Representative Concentration Pathways-Shared Socioeconomic Pathway (RCP-SSPs) scenarios from 2020 to 2100. Deep blue, light blue, orange, and deep red represent SSP1-2.6, SSP2-4.5, SSP3-7.0, and SSP5-8.5 scenarios, respectively. The black line indicates the average trajectory. Two extreme results by the end of the century are highlighted: the highest from FGOALS-g3 under SSP1-2.6 and the lowest from MIROC-ES2L under SSP5-8.5. (B) Ratio of actual scenario to ideal scenario EV electricity demand compared to the level in 2050 (left box with gray border) versus 2100 (right box with black border) across countries. Each round of prediction (shown as diamond-shaped points) utilizes data simulated from 11 ensemble GCMs under four RCP-SSP scenarios. Country abbreviations correspond to the ISO 3166-1 alpha-2 standard (refer to Table S7). (C–E) Ideal electricity demand globally by the end of this century (C), the ratio of additional EVs electricity demand by ambient temperature (D), and the relative change in this demand ratio compared to the mid-century (E) under four RCP-SSP scenarios. All projections here are based on actual (with ambient temperature) and ideal (without ambient temperature) scenarios.

To distinguish between climate change effects, we analyze both relative temperature sensitivity and absolute electricity demand trends. We compare two scenarios throughout: the actual scenario (considering ambient temperature) and the ideal scenario (excluding ambient temperature). We define the baseline as an idealized scenario without temperature effects. All results here reflect the deviation from this baseline due solely to temperature-driven electricity use. In the coming decades, while the global electricity demand for EVs is expected to rapidly increase (see detailed data in Table S8), the impact of ambient temperature on EVs’ electricity consumption is projected to decrease (Figure 5A; see detailed data in Table S9). As shown in Figure 5B, on average across countries, the impact of ambient temperature on electricity demand for EVs is projected to decrease by about 1.4% from 11.4% in 2050 to 10.0% in 2100. Canada is expected to experience the largest decline from 30% in 2050 to 24% in 2100, decreasing by 6%. However, countries in tropical and subtropical regions, such as South Africa, Australia, Israel, Mexico, India, and Brazil, are expected to experience additional EVs’ electricity consumption increases of 0.02%–1.07%. This trend can be attributed to the more frequent occurrence of hot weather in these nations, leading to battery overheating and increased power demand for vehicle cooling systems. Except for the United States and China, countries where electricity consumption has alleviated are mostly located in higher latitudes of the Northern Hemisphere. These countries have historically experienced colder climates and longer winters, experiencing more pronounced warming under global climate change.^36^

Figure 5C shows the differences in ideal versus actual electricity consumption across various regions globally under different scenarios. North America, Europe, and East Asia consistently exhibit the highest electricity demand for EVs. Relative to the ideal scenario, global warming has alleviated the impact of temperature on EVs’ electricity use. Figures 5D and 4E indicate that, under the SSP1-2.6 scenario, only some regions in the Arctic Circle, Greenland, and Canada can directly benefit from global warming, leading to a significant reduction in EVs’ additional electricity demand, whereas Europe, Northern Asia, and Siberia would experience an increase in electricity consumption. As for certain regions in North America, Europe, and Asia, some benefit from an overall increase in vehicle air conditioning demand due to climate warming, while others benefit from milder ambient temperatures. Tropical and equatorial regions show minimal changes, suggesting lower sensitivity of EVs to temperature variations compared to temperate and polar regions. Under the SSP2-4.5 scenario, more regions will experience pronounced climate warming, especially in the Northern Hemisphere, such as the northern North America and the Arctic region, leading to a decrease in electricity consumption driven by ambient temperature. However, other regions in the Northern Hemisphere, such as parts of Africa, Asia, southern United States, and northern South America, will suffer an increase in additional electricity demand due to higher cooling and vehicle air conditioning needs. Under the SSP3-7.0 scenario, the mid-latitude regions of the Northern Hemisphere and the southern regions of South Africa, Chile, Argentina, and Peru will witness decreased power consumption due to fewer extreme cold weather events. Meanwhile, low-latitude regions near the equator can experience a significant increase in electricity consumption due to more frequent heatwaves and higher cooling demands. Under the SSP5-8.5 scenario, a higher radiative forcing setting, significant decreases in electricity demand are expected in North America, Northern Europe, Northern Asia, and the Arctic region due to milder winters and reduced heating demands. Temperate regions like central North America, mid-latitude Europe, and Asia, show slight increases or decreases in electricity demand due to subtle climate changes in summer and winter. Most regions in the Middle East, South Asia, Australia, and Africa witness an increase in additional electricity demand driven by more frequent heatwaves and sustained high temperatures under global warming.^35^

## Discussion

Our analysis shows that ambient temperature has a notable impact on electricity consumption by EVs. Despite the rapid rise in total electricity demand from EVs over the coming decades, expected to increase 40 times by 2050, the relative temperature impact (measured as percentage deviation from ideal operating conditions) decreases from 12.3% in 2030 to 10% by the end of this century. This demonstrates that climate change reduces the relative temperature sensitivity of the global EV fleet, primarily due to the reduction of extreme cold days that stress EV batteries and range. However, there are notable differences in the patterns of change across regions, with areas near the equator and parts of the Southern Hemisphere seeing temperature-driven electricity consumption rise due to increased cooling needs.

The substantial and uneven impact of ambient temperature on EV electricity use has important implications for climate change mitigation and energy system planning. On the one hand, our results suggest that as winters become milder, concerns about substantial reductions in EV range and efficiency in cold weather can diminish. This could make EVs more attractive to consumers and accelerate fleet electrification. On the other hand, the remaining 10% of electricity demand driven by temperature by 2100, particularly cooling needs in hot climates, still represents a vast amount of electricity—over 310 TWh in that year alone. If this electricity is supplied through carbon-intensive generation, it would result in substantial GHG emissions, discounting the effectiveness of EVs as a mitigation strategy. Managing this temperature-sensitive load through renewable energy, power storage, smart charging, and resilient transmission can provide a solution.^24^^,^^37^

### Limitations of the study

There are several limitations in this study that warrant future work. We acknowledge that battery aging and degradation are not modeled, which may underestimate long-term temperature impacts on energy consumption. While we downscale country-level EV projections to the grid-level based on population and economic factors, we cannot capture all determinants of local vehicle adoption patterns such as charging infrastructure availability, electricity grid capacity, vehicle pricing differences, and localized policy incentives. These factors likely create heterogeneity in adoption that our model cannot fully represent. Our EV model training data come from California, which has well-developed charging network and strong incentive programs create favorable conditions for EV adoption, potentially leading to overestimation in regions with infrastructure constraints.^38^ While temperature is the primary variable modeled, other unobserved factors such as driving behavior and road conditions may also influence energy use and are partially embedded in the data-driven estimates. In addition, EV technical efficiency is modeled using a standard exponential decay form, calibrated with limited data points from policy reports, serving as a plausible approximation rather than a detailed empirical reconstruction. Uncertainties in warming and socioeconomic trajectories based on General Circulation Models (GCMs) also widen the range of uncertainties. Future work should incorporate spatially explicit data on charging infrastructure deployment and policy implementation when such global datasets become available. Additionally, quantifying the carbon implications of temperature-sensitive EV demand across evolving energy systems represents an important research direction. Overcoming these limitations requires expanded data collection and modeling innovation. Nevertheless, the consistency of our findings across scenarios and with more granular analyses enhances confidence in the overall trends and magnitudes reported.

## Resource availability

### Lead contact

Further information and requests for resources and reagents should be directed to and will be fulfilled by the lead contact, Yifang Zhu, Ph.D. (yifang@ucla.edu).

### Materials availability

Materials generated in this study, including model running results, can be requested from the lead contact.

### Data and code availability

The primary data supporting our research findings are as follows. (1) Global historical monthly high-resolution meteorological data are available from TerraClimate (https://www.climatologylab.org/datasets.html) and the Climatic Research Unit database (https://crudata.uea.ac.uk/cru/data/hrg/). (2) EV data at the census tract level in California are sourced from the California Air Resources Board and the U.S. Census Bureau (https://arb.ca.gov/emfac/fleet-db/e47017fbfbc354ea477543661846e8a5728e3f32). Population, GDP, and administrative area information for California are obtained from the Master Address File/Topologically Integrated Geographic Encoding and Referencing Database (https://catalog.data.gov/dataset/tiger-line-shapefile-2019-state-california-current-census-tract-state-based) and Wang and Sun (2023) (https://zenodo.org/records/7898409).^39^ (3) Historical and projected EV stock, energy use, and related policy developments for global countries and regions are sourced from the Electric Vehicles Initiative (https://www.iea.org/data-and-statistics/data-tools/global-ev-data-explorer). (4) GDP and population data at a spatial resolution of 0.5° for the years 2020–2100 under five Shared Socioeconomic Pathways are obtained from Jiang et al. (2024).^40^ (5) The per-vehicle mileage and electricity efficiency functions of EVs are primarily referenced from Hou et al. (2021) and the EV Outlook series.^41^ (6) Data from 11 General Circulation Models under four Representative Concentration Pathways-Shared Socioeconomic Pathways are sourced from the Coupled Model Intercomparison Project Phase 6 (https://esgf-node.llnl.gov/search/cmip6/). The data files, model outputs, and a key Python script supporting the analyses presented in the publication have been uploaded to https://zenodo.org/records/15755862.

## Acknowledgments

This work was supported by 10.13039/100014576University of California Office of the President Climate Action under grant number R02CP6948.

## Author contributions

Study conception and design, Q.W. and Y.Z.; data collection, Q.W.; analysis and interpretation of the results, Q.W.; paper drafting, preparation, and revision, Q.W. and Y.Z. All authors reviewed the results and approved the final version of the paper.

## Declaration of interests

The authors declare no competing interests.

## STAR★Methods

### Key resources table

**Table:** 

REAGENT or RESOURCE	SOURCE	IDENTIFIER
**Deposited data**		

Global historical monthly high-resolution meteorological data	TerraClimate; Climatic Research Unit (CRU)	https://www.climatologylab.org/datasets.html; https://crudata.uea.ac.uk/cru/data/hrg/
EV data at the census tract level in California	California Air Resources Board; US Census Bureau	https://arb.ca.gov/emfac/fleet-db/e47017fbfbc354ea477543661846e8a5728e3f32
Population, GDP, and administrative area information for California	MAF/TIGER Database; Wang and Sun (2023)^39^	https://catalog.data.gov/dataset/tiger-line-shapefile-2019-state-california-current-census-tract-state-based; https://zenodo.org/records/7898409
Historical and projected EV stock and energy use for global countries	Electric Vehicles Initiative (IEA)	https://www.iea.org/data-and-statistics/data-tools/global-ev-data-explorer
Projected GDP and population data (0.5° resolution, 2020–2100, under SSPs)	Jiang et al.^40^	See reference in manuscript
Per-vehicle mileage and electricity efficiency functions	Hou et al.^41^; EV Outlook series (IEA)	See references in manuscript
Future climate data from 11 GCMs (under four RCP-SSPs)	Coupled Model Intercomparison Project Phase 6 (CMIP6)	https://esgf-node.llnl.gov/search/cmip6/
Processed data and model outputs generated in this study	This paper; Zenodo	https://zenodo.org/records/15755862

**Software and algorithms**		

Python (Version 3.13.5)	Python Software Foundation	https://www.python.org/
Jupyter Notebook	Project Jupyter	https://jupyter.org/
R (Version 4.5.1)	The R Foundation for Statistical Computing	https://www.r-project.org/
ArcGIS Pro	Esri	https://www.esri.com/arcgis/products/arcgis-pro
Google Earth Engine	Google	https://earthengine.google.com/

### Method details

To assess the influence of ambient temperature on EV electricity consumption across global regions and future climate scenarios, we developed an integrated methodological framework as illustrated in Figure 5. Our approach integrates multiple data sources and analytical techniques through five interconnected tasks. We established the temperature-dependent electricity consumption response function for EVs (*Task 1*) and predicted future EV stock distribution at high spatial resolution (*Task 2*). Vehicle travel distances (*Task 3*) and technical efficiency improvements (*Task 4*) were estimated to calculate electricity demand under both ideal and actual temperature conditions. Temperature effects on electricity consumption from 2010 to 2100 were simulated using 11 global climate models across four SSPs (*Task 5*). This comprehensive framework enables systematic assessment of ambient temperature effects on EV electricity consumption in a warming world. The following sections detail the specific methods employed for each component.

#### Fitting the nonlinear response function of EV’s electricity consumption to ambient temperature

We outline a four-step process for estimating the response function of EV electricity consumption to ambient temperature for each 0.5 × 0.5° grid.

**Step 1:** We perceive extra EV electricity intensity (EVEI) as a function of temperature T’s probability distribution:

(Equation 1) $$\begin{array}{c} EVEI\left(T\right)=EVEI\left(\omega \left(t\right)\right)=\int_{-\infty }^{+\infty }f\left(t\right)\omega \left(t\right)dt=\beta^{-\alpha }\int_{-\infty }^{t_{\alpha }}\omega \left(t\right)dt+\beta^{\alpha +}\int_{t_{\alpha }}^{+\infty }\omega \left(t\right)dt\\ f\left(t\right)=\left\{ \begin{array}{c} \beta^{-\alpha }\;\text{if}\;t\in \left(-\infty ,t_{\alpha }\right)\\ \beta^{\alpha +}\;\text{if}\;t\in \left(t_{\alpha },+\infty \right) \end{array}\right. \end{array}$$

(Equation 2) $$\begin{array}{c} EVEI\left(T\right)=\lim_{n\to \infty }\lim_{\Delta t\to 0}\sum_{l=1}^{n}\int_{t_{l}}^{t_{l}+\Delta t}\beta_{n}\left(t-t_{\alpha }\right)\omega \left(t\right)dt \end{array}$$

in which, *f*(*t*) and *ω*(*t*) respectively represent the probability density functions of EV electricity intensity and temperature distribution controlled by the instantaneous time. The selection of the quantile *α* is arbitrary, taking the example of the optimal temperature *t*_*α*_ for EVEI. When *t* < *t*_*α*_, *f*(*t*) = *β*^*-α*^ > 0, while when *t* > *t*_*α*_, *f*(*t*) = *β*^*α+*^ > 0. The difference between *β*^*α*+^ and *β*^*-α*^ is the gradient of EVEI in this temperature range. The integral of *ω*(*t*) at the two ends of *t*_*α*_ represents the corresponding occurrence probability. Equations 1 and 2 highlight that the influence of temperature distribution on EVEI depends on the specific intervals. Combining real monthly average temperatures, we set the interval *Δt* of temperature ranges to 3 °C, dividing the temperature distribution into 36 intervals These intervals range from temperatures below −48 °C to those above 57 °C, with specific intervals including [−48 °C, −45 °C), [−45 °C, −42 °C), …, [57 °C, 60 °C).

**Step 2:** Considering the optimal temperature for EV operation as non-time-varying, we assume it depends on the main meteorological factors. We obtain maximum and minimum temperature, precipitation, solar radiation, vapor pressure, and wind speed, from TerraClimate at 0.5 × 0.5° resolution and calculate their monthly averages respectively during 2015–2020.^42^

**Step 3:** To determine the optimal temperature, we conduct a meta-analysis of existing literature examining how EV electricity consumption responses to ambient temperature. This meta-analysis incorporates data from several peer-reviewed studies and reports, covering over 5 million trips across diverse climates, vehicle types, and regions. Here, “optimal temperature” refers to the temperature with minimum observed consumption. Its regional variation reflects not only temperature effects but also location-specific factors implicitly captured in the data. We identify the most representative fleet survey from Argue as the benchmark, which analyzes 5.2 million trips from 4,200 EVs covering 102 make–model–year combinations across global climates.^7^ This single comprehensive study formed our primary reference point. And the data reflect real-world driving conditions and reveal a consistent temperature-efficiency curve, peaking at ∼21.5 °C, with range dropping significantly in both cold and hot extremes.^7^ From this dataset, we calculate the difference between the optimal temperature reported by other studies in our meta-analysis and their optimal temperature, termed as the first-order variation, serving as the target variable for our machine learning model. Utilizing the experimental locations stated in the literature to obtain the geographic coordinates and meteorological conditions of those areas. We employ a feature-selected random forest model to predict the first-order offset of the optimal temperature, with a 0.5 × 0.5° resolution. Finally, we combine these predictions with benchmark to determine the optimal temperature.

**Step 4:** For each response curve in the meta-analysis literature, we divide the temperature range into 3 °C intervals and calculate the additional electricity consumption relative to the optimal temperature. We then calculate the difference of this increase from the benchmark, termed as the first-order variation, which served as the target variable for the machine learning model. Subsequently, utilizing the meteorological factors and the derived optimal temperature in Steps 2 and 3 as input variables, we employed a feature-selected random forest model for prediction. This process yielded the response pattern of EVEI to temperature gradients at a resolution of 0.5 × 0.5° for each grid. Consequently, we obtained the electricity consumption ∇EVEI(*T*) for EVs in different temperature intervals.

#### Forecasting future electric vehicles for electric vehicle stocks across countries

The IEA and the EVs Initiative provide key areas of interest such as EV stock, energy use, battery demand and related policy developments in 33 regions such as the United States, China, India, as well as the Rest of the World, from 2010 to 2022. Additionally, projections for the years 2025 and 2030 are available for the United States, China, Europe, India, and the Rest of the World. These projections account for specific EV policies, categorized into STEPS and APS. STEPS represents a scenario where only currently declared policies and measures are considered, whereas APS incorporates the potential impact of more ambitious policy objectives, larger emission reduction targets, or assumptions regarding technological advancements that countries or regions may announce but have yet to implement (defined in Table S10). It’s important to note that EVs are categorized into two main types: BEVs and PHEVs. BEVs solely rely on battery power, while PHEVs combine both battery and internal combustion engine power. Our methodology draws upon the framework established by Hou et al.^41^ for determining the peak time of EV stock. Curve setting follows the logistic function (Equation 3) and is optimized using the differential evolution algorithm (Equation 4), we trained our models based on historical EV data spanning from 2010 to 2022. Subsequently, we generate four sets of forecasted stock estimates for BEVs and PHEVs under both STEPS and APS for the years 2023–2100:

(Equation 3) $$\begin{array}{c} {{EVStock}^{Cap}}_{c}^{y,p}\left(t\right)=\frac{K}{1+{\left(\frac{K-P_{0}}{P_{0}}\right)e}^{-rt}} \end{array}$$

(Equation 4) $$\begin{array}{c} s.t.\;minimize\sum_{i=1}^{n}{\left({{{EVStock}^{Cap}}_{c}^{y,p}\left(t_{i}\right)}_{Trained}-{{{EVStock}^{Cap}}_{c}^{y,p}\left(t_{i}\right)}_{Observed}\right)}^{2} \end{array}$$

in which, *c*, and *t* individually represent countries, and years; *y* and *p* respectively denote the two types of EVs (BEV and PHEV) and the two policy scenarios (STEPS and APS).

#### Machine learning framework for downscaling electric vehicles for electric vehicle stocks

We choose California’s EV data as the training set for several reasons: 1) California is one of the leaders in EV markets among the United States and worldwide, boasting the largest stock and sales volume of EVs. 2) California’s statistics are more comprehensive, accurate, and diverse, providing rich and varied data for modeling. 3) The climate conditions in California are conducive to EV operation. Falling within mild ambient conditions favors battery performance and range, thereby enhancing our machine learning model’s training and generalization capabilities.^18^^,^^30^

We obtain census tract-level vehicle population data from the California Air Resources Board for the period 2015 to 2020.^43^ Population for each census tract are obtained from the US Census Bureau. Leveraging the map polygons provided by the US Census Bureau’s Master Address File/Topologically Integrated Geographic Encoding and Referencing (MAF/TIGER) Database (MTDB),^39^,^44^ we calculated the administrative area and GDP across census tract using the 1 × 1 km gridded GDP data.^39^

We utilize the logarithm of population, GDP, and administrative area, incorporating their linear, quadratic, and general interaction terms (including pairwise, three-way, and the quadratic forms of these interactions) as feature variables. The logarithm of the EV stock is used as the outcome variable. Employing a feature-selected random forest model, as depicted in Equation 5, the training set consisted of 38,659 observations from 2015 to 2019, while the validation set comprised 6,824 observations in 2020:

(Equation 5) $$\begin{array}{c} {EVStock}_{cs,t}^{RF}=RF\left(G,P,A,GP,GA,PA,GPA\right)+\epsilon_{cs,t}^{RF} \end{array}$$

in which, *t* represents the year. *G*, *P*, and *A* respectively denote the GDP, population, and administrative area of a census tract (cs).

Additionally, we acknowledge that using primarily population and GDP for downscaling is a simplification necessitated by global data availability. Local factors such as charging infrastructure density, electricity grid capacity, vehicle prices, and policy incentives also influence adoption patterns. However, consistent global datasets for these variables at 0.5° resolution are currently unavailable. Our validation against California data, where these factors are relatively favorable for EV adoption, shows good predictive performance, but may overestimate adoption in regions with underdeveloped infrastructure or limited incentives.

#### Downscaling total electric vehicles for electric vehicle stocks to grid-level resolution

Jiang et al. utilized the Population Development Environment model and the Cobb-Douglas model to provide GDP and population data at a 0.5° spatial resolution for the years 2020–2100, under five SSPs.^40^ The land surface area of each grid is identified using Google Earth Engine. Using the GDP, population, and administrative area, along with their feature engineering as same as above, we input each pixel into the acquired machine learning framework and obtain the EV stock ${{EVStock}^{ML}}_{ij,c,t}^{s,y,p}$. Utilizing this as a weight and combining it with the EV stock ${{EVStock}^{Pred}}_{ij,c,t}^{s,y,p}$ obtained earlier for two types of EVs and two EV policy scenarios for the years 2023–2100, we derive the predicted EV stock ${{EVStock}^{Pred}}_{ij,c,t}^{s,y,p}$:

(Equation 6) $$\begin{array}{c} {{EVStock}^{Pred}}_{ij,c,t}^{s,y,p}={{EVStock}^{Cap}}_{c,t}^{y,p}\times \frac{{{EVStock}^{ML}}_{ij,c,t}^{s,y,p}}{\sum {{EVStock}^{ML}}_{ij,c,t}^{s,y,p}} \end{array}$$

in which, *ij*, *c*, and *t* respectively represent the GIS coordinates (*i*, *j*) of each grid, countries, and years; *s*, *y*, and *p* respectively denote the five SSPs, two types of EVs, and two policy scenarios.

#### Calculating annual vehicle-miles traveled per electric vehicles for electric vehicle

EV VMT is linked to the economic prosperity measured by per capita income.^41^ In regions characterized by EMDEs, the burgeoning per capita GDP in recent years has spurred an uptick in VMT per EV. In the more matured territories of North America, Europe, and Oceania, where GDP expansion has plateaued and travel demands have plateaued, the growth in per capita mileage has either stagnated or exhibited a sluggish trajectory. We model the per capita mileage following a cumulative Weibull function with the per capita GDP:^41^^,^^45^

(Equation 7) $$\begin{array}{c} {EVTraveled}_{ij,\tau ,t}^{s}={EVTraveled}_{ij,\tau ,t}^{*}\times \left(1-e^{-{\left(\frac{r_{\tau }\left(t\right)-r_{\tau ,0}}{10000}\right)}^{\sigma }}\right) \end{array}$$

where *σ* represents the shape parameter of the Weibull function; $r_{\tau }\left(t\right)$ denotes the per capita GDP of the τ-th continent in year *t*; $r_{\tau ,0}$ is the initial per capita GDP of the *τ*-th continent when significant increases in car travel distance commence; and ${EVTraveled}_{ij,\tau ,t}^{*}$ signifies the saturated per capita car travel distance. The saturated per capita car travel distance is influenced by the geographical, road infrastructure, public transportation, and traffic policy levels of each continent, as well as subsequent multi-round calibrations. The shape parameter, saturated per capita car travel distance, and initial per capita GDP are all fitted based on previous studies.^41^

#### Estimating EV’s technical efficiency function

The technical efficiency of EVs, measured in kWh per kilometer (kWh/km), is contingent upon innovations in battery technology. EV related technology, being a public good under international trade, is driven by global automotive manufacturers and technology providers, facilitating rapid diffusion and widespread adoption across various regions.^29^^,^^46^ On an annual scale, technical efficiency can remain consistent across all regions. The temporal evolution of EV technical efficiency (EVTE) follows an exponential decay learning curve, indicating rapid initial technological advancements followed by a gradual slowdown in the rate of change.

(Equation 8) $$\begin{array}{c} {EVTE}_{t}=\alpha +e^{-\beta \times \left(t-2000\right)}+\theta \end{array}$$

here, the variable *t* denotes the year; parameters *α*, *β*, and *θ* are derived from data fitting based on comprehensive datasets sourced from the Fuel Economy Guide by the United States Environmental Protection Agency spanning from 2011 to 2017,^47^ the Global EV Outlook series reports by the IEA for the year 2018, 2019, and 2020,^48^^,^^49^^,^^50^ its Energy Technology Perspectives report in 2017,^46^ and EV Database.^51^

As illustrated in Figure S2, the upper limit of the technical efficiency curve is derived from data extracted from the Global EV Outlook reports for 2018 and 2019,^48^^,^^50^ fitted utilizing Equation 8, whereas the lower limit curve is established through fitting based on the left-sided data points. This functional form is widely adopted in long-term energy modeling, representing the typical trend of technological learning.^41^ Each year in the lower-limit curve corresponds to the average combined-cycle energy intensity across all reported EV models in the EPA’s Fuel Economy Guide, without separating vehicle size classes. The baseline technical efficiency corresponds to the mean value between the upper and lower limits. This study does not incorporate battery degradation or lifetime aging effects; all modeled electricity consumption reflects instantaneous temperature impacts.

#### Estimating electric vehicles for electric vehicle electricity consumption

The monthly average temperature serves as a proxy to capture the variability of seasons and extreme weather conditions. We utilize monthly average temperature data to calculate electricity consumption, while aligning other annual variables through linear interpolation to match the monthly scale. Finally, we aggregate the monthly electricity consumption of EV to an annual level.

In the ideal scenario without considering temperature factors, the electricity consumption of EVs, denoted as ${EVEC}^{WO/TEM}$, depends on the three factors: EV stock, VMT, and electricity efficiency, as shown in Equation 9. However, in actual scenarios influenced by ambient temperature, the electricity consumption of EVs can be calculated incorporating temperature function ∇EVEI(T), as depicted in Equation 10:

(Equation 9) $$\begin{array}{c} {{EVEC}^{\frac{WO}{TEM}}}_{ij,c,t}^{s,y,p,G}=\sum_{m=1}^{12}{EVStock}_{ij,c,m,t}^{s,y,p}\times {EVTE}_{m,t}\times {EVTraveled}_{ij,m,t}^{s} \end{array}$$

(Equation 10) $$\begin{array}{c} {{EVEC}^{\frac{WO}{TEM}}}_{ij,c,t}^{s,y,p,G}=\sum_{m=1}^{12}\left[1+{\nabla EVEI\left(T\right)}_{ij,c,m,t}^{s,y,p,G}\right]\times {EVStock}_{ij,c,m,t}^{s,y,p}\times {EVTE}_{m,t}\times {EVTraveled}_{ij,m,t}^{s} \end{array}$$

in which, *ij* represents the GIS coordinates (*i*, *j*) of each grid, *c* denotes a country, *m* signifies the month, *t* indicates the year of that month, *s*, *y*, and *p* respectively represent five SSPs, two types of EVs, and two policy scenarios. *G* stands for 11 GCMs.

#### Scenario settings for electric vehicles for electric vehicle electricity demand

We leveraged economic, demographic, and temperature data across four RCP-SSPs scenarios from 2010 to 2100, sourced from 11 representative GCMs from the Coupled Model Intercomparison Project Phase 6.^35^ These scenarios encompassed shifts in economic and population development levels, travel behaviors, advancements in energy technology, and changes in policy directions.

**Scenario set 1: Historical Ideal Scenario.** Using Equation 9, we examine the electricity consumption of EVs from 2010 to 2022 without considering ambient temperature factors, denoted as ${EVEC}^{WO/TEM}$.

**Scenario set 2: Historical Actual Scenario.** Utilizing Equation 10, we investigate the electricity consumption of EVs from 2010 to 2022 when considering ambient temperature variations, denoted as ${EVEC}^{W/TEM}$.

**Scenario set 3: Future Scenarios.** We select 11 representative GCMs from Phase 6 of the Coupled Model Intercomparison Project and extract the monthly average temperature data from 2020 to 2100 under four typical RCP-SSPs scenarios. The spatial resolution for all GCMs is downscaled to 0.5 × 0.5° resolution. Detailed information regarding the GCMs, SSP, and RCP-SSP scenarios can be found in Tables S11–S13, respectively. For each specific RCP-SSP pairing, we coupled it with corresponding global EV stock (*EVStock*) and EV Traveled (*EVTraveled*) data. Subsequently, EV electricity consumption is computed using Equations 9 and 10.

#### Electric vehicles for electric vehicle electricity consumption due to ambient temperature

Equations 11 and 12 are both used to calculate the increase in EV electricity consumption attributed to ambient temperature in the ideal and actual scenarios denoted as ${EVEC}^{TEM}$:

(Equation 11) $$\begin{array}{c} {{EVEC}_{A}^{TEM}}_{ij,c,t}^{s,y,p,G}={{EVEC}^{W/TEM}}_{ij,c,t}^{s,y,p,G}/{{EVEC}^{WO/TEM}}_{ij,c,t}^{s,y,p,G} \end{array}$$

or

(Equation 12) $$\begin{array}{c} {{EVEC}_{B}^{TEM}}_{ij,c,t}^{s,y,p,G}={{EVEC}^{W/TEM}}_{ij,c,t}^{s,y,p,G}-{{EVEC}^{WO/TEM}}_{ij,c,t}^{s,y,p,G} \end{array}$$

#### Aggregating electric vehicles for electric vehicle electricity consumption from grid-level to country-level

By utilizing area integration, we aggregate grid variables to the national level, where *S*_*ij*_ represents the area of grid *ij* under 0.5 × 0.5° resolution, and *Y*_*ij*_ represents the variable value on that grid.

(Equation 13) $$\begin{array}{c} Y_{c,t}^{s,y,p,G}=\iint_{\Omega_{c}^{-}}^{\Omega_{c}^{+}}Y\left(i,j\right)dS=\sum_{i=i,j=j}Y_{i,j,c,t}^{s,y,p,G};\left(i,j\right)\in \Omega_{c} \end{array}$$

where *ij* represents a grid at coordinate (*i*,*j*), *c* denotes a country, and *t* denotes the year of that month. Y(*i*,*j*) denotes the variable value at grid *ij*. *dS* represents a small area element of 0.5 × 0.5°. *Ω*_*c*_ represents the spatial extent of country c, indicating that the integration is conducted within this region. *s*, *y*, and *p* respectively denote four RCP-SSPs pathways, two types of EVs, and two policy scenarios. G represents eleven GCMs.

### Quantification and statistical analysis

The random forest model for downscaling EV stocks achieved an R^2^ of 0.69, RMSE of 0.27, MAE of 0.17, and MSE of 0.07. Residuals are symmetrically distributed with no strong heteroscedasticity, and feature importance analysis indicates that population and population–area interactions are dominant predictors.

In this study, sensitivity analysis is conducted based on a multi-scenario, multi-model framework. We account for uncertainty in our overall conclusions across seven dimensions: (1) Different population-economic development patterns under various RCPs and SSPs; (2) Variability in monthly average temperature outputs from different GCMs; (3) Future national-level EV stocks under different policy scenarios; (4) Different types of EVs affected; (5) Expected impact of EV battery technology on their energy efficiency; (6) Annual driving distance per EV under different RCP-SSPs. We predict the distribution of EV electricity demand by ambient temperature with altering different parameters (e.g., population and GDP in the first group, monthly average temperature in the second group, EV policy backgrounds in the third group, types of EVs in the fourth group, technical efficiency parameters of EVs in the fifth group, annual driving distance per EV in the sixth group) to enhance the robustness of our conclusion.
