# Supplementary material for: Effects of ambient temperature on electric vehicle electricity consumption are reduced in a warming world
Source: iScience. 2025 Aug 27;28(9):113328. doi: 10.1016/j.isci.2025.113328 (PMC12496165; doi:10.1016/j.isci.2025.113328)
Supplement: Document S1. Figures S1, S2, and Tables S1–S13 [file mmc1.pdf]

iScience, Volume *28*

Supplemental information

Effects of ambient temperature on electric vehicle electricity consumption are reduced in a warming world

Qingyang Wu and Yifang Zhu

Supplementary Information

This supplementary information includes **Figures S1-S2,** and **Tables S1-S13**.

Supplementary Figures

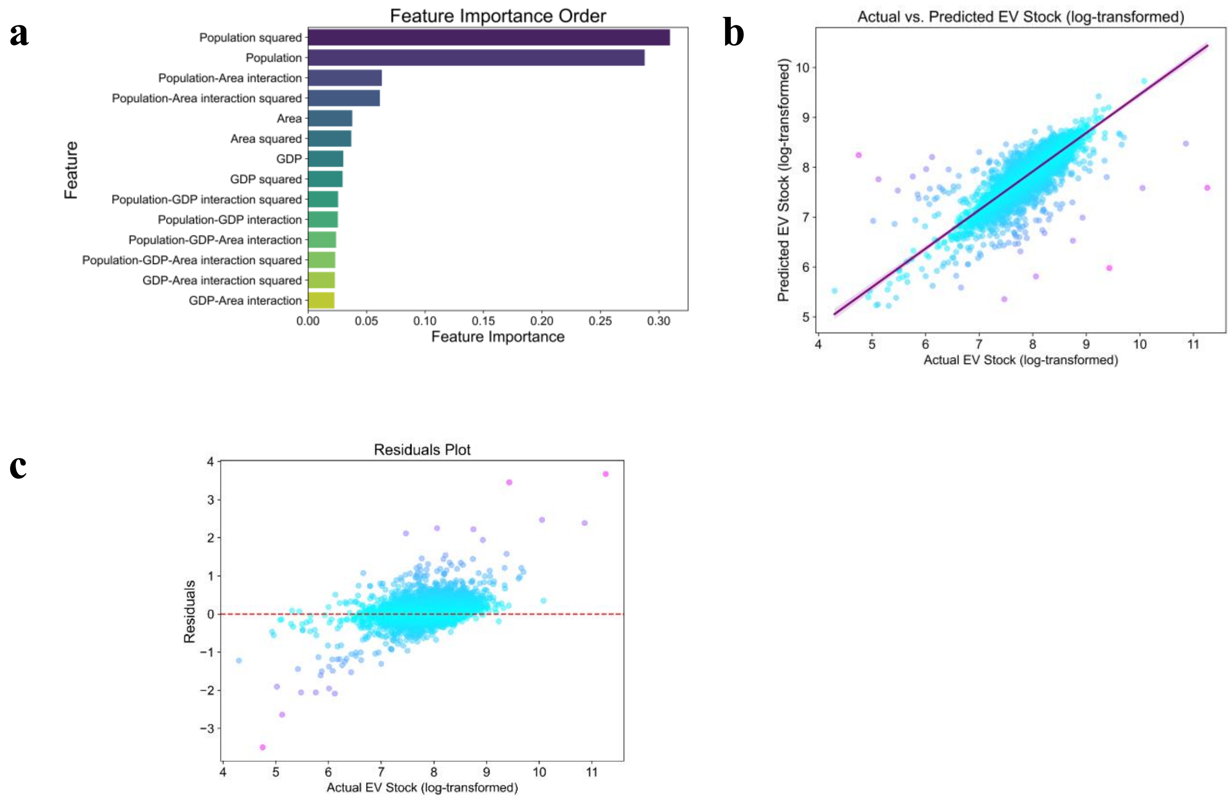

Figure S1 | Predictive results and feature importance in machine learning. a The importance of each feature variable is displayed by order, arranged from top (purple) to bottom (yellow). **b** The fit line and scatter plot of the actual 2020 EV stock values against the model's predicted values for 2020, depicted with purple lines and blue dots, respectively. **c** The residuals plot of actual versus predicted 2020 EV stock values, with dot colors from blue to purple representing increasing deviation from zero.

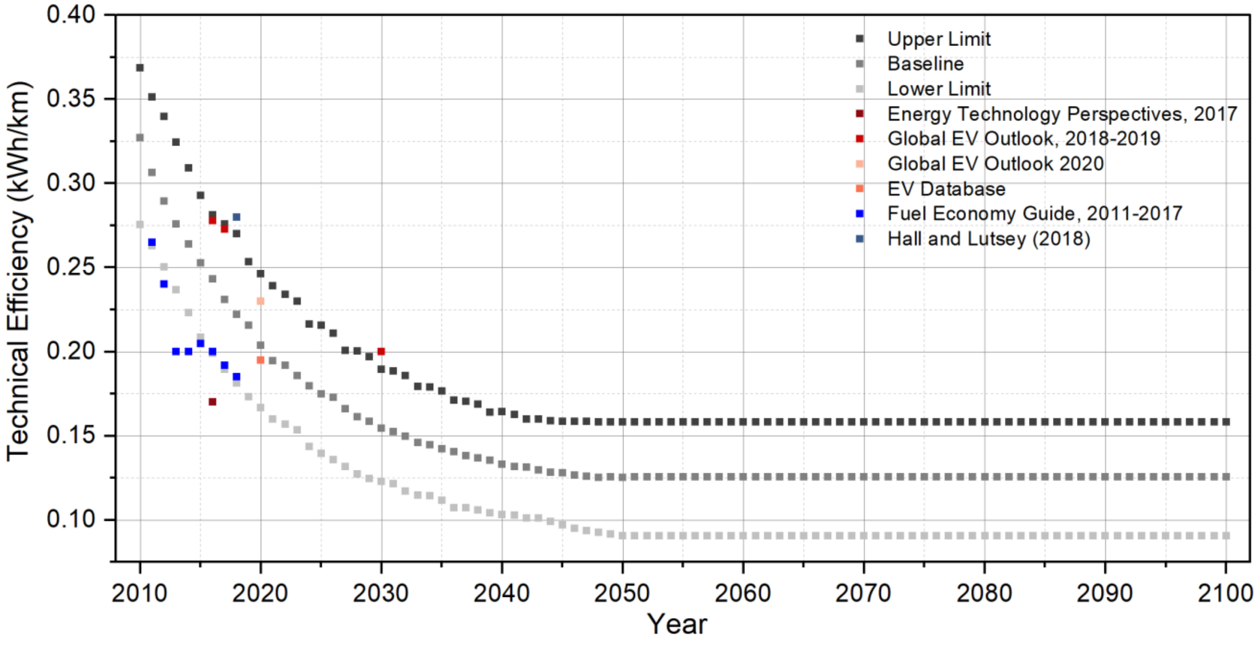

Figure S2 | EV's technical efficiency functions from policy documents (2010-2100). This graph depicts the energy intensity (kWh/km) from 2010 to 2100, with various sources represented by different colors: black (upper limit), dark grey (baseline), light grey (lower limit), red (Energy Technology Perspectives, 2017),[1] dark red (Global EV Outlook 2018 and 2019),[2,3] light red (Global EV Outlook, 2020),[4] grey (EV Database),[5] blue (Fuel Economy Guide, 2011-2017),[6] and blue dashes (Hall and Lutsey, 2018).[7] This figure combines theoretical learning curves with empirical point-based calibration, following the modeling framework introduced by Hou *et al*.[8]

Supplementary Tables

Table S1 | BEV stock across 33 major countries and regions (includes the "Rest of the World") under STEPS from 2010 to 2100 (units: million vehicles).

Name	2020	2021	2022	2025	2030	2035	2040	2045	2050	2055	2060	2065	2070	2075	2080	2085	2090	2095	2100
Australia	0.02	0.03	0.07	0.23	1.08	2.49	3.11	3.23	3.25	3.25	3.25	3.25	3.25	3.25	3.25	3.25	3.25	3.25	3.25
Austria	0.05	0.08	0.12	0.32	1.00	2.05	2.75	2.99	3.06	3.08	3.08	3.08	3.08	3.08	3.08	3.08	3.08	3.08	3.08
Belgium	0.03	0.05	0.09	0.25	0.81	1.67	2.22	2.40	2.45	2.46	2.46	2.46	2.46	2.46	2.46	2.46	2.46	2.46	2.46
Brazil	0.00	0.00	0.01	0.07	0.13	0.24	0.39	0.54	0.67	0.74	0.78	0.80	0.81	0.82	0.82	0.82	0.82	0.82	0.82
Canada	0.11	0.16	0.26	0.93	4.23	9.67	12.15	12.66	12.75	12.76	12.76	12.76	12.76	12.76	12.76	12.76	12.76	12.76	12.76
Chile	0.00	0.00	0.00	0.01	0.02	0.03	0.05	0.07	0.08	0.09	0.09	0.10	0.10	0.10	0.10	0.10	0.10	0.10	0.10
China	4.46	7.30	12.42	30.70	59.11	104.76	164.65	225.29	272.57	302.45	318.92	327.31	331.42	333.39	334.32	334.77	334.97	335.07	335.12
Denmark	0.03	0.07	0.12	0.30	0.99	2.04	2.71	2.94	2.99	3.01	3.01	3.01	3.01	3.01	3.01	3.01	3.01	3.01	3.01
Finland	0.01	0.02	0.05	0.12	0.41	0.85	1.12	1.21	1.23	1.23	1.23	1.23	1.23	1.23	1.23	1.23	1.23	1.23	1.23
France	0.34	0.51	0.70	1.93	5.87	11.94	16.08	17.60	18.03	18.14	18.16	18.17	18.17	18.17	18.17	18.17	18.17	18.17	18.17
Germany	0.36	0.73	1.06	2.80	9.03	18.68	24.83	26.89	27.42	27.55	27.58	27.58	27.58	27.59	27.59	27.59	27.59	27.59	27.59
Greece	0.00	0.00	0.01	0.02	0.07	0.14	0.18	0.20	0.20	0.20	0.20	0.20	0.20	0.20	0.20	0.20	0.20	0.20	0.20
Iceland	0.01	0.01	0.02	0.05	0.16	0.33	0.44	0.47	0.48	0.49	0.49	0.49	0.49	0.49	0.49	0.49	0.49	0.49	0.49
India	0.02	0.03	0.08	1.04	2.12	3.97	6.56	9.30	11.44	12.77	13.47	13.81	13.97	14.05	14.08	14.10	14.10	14.11	14.11
Israel	0.00	0.02	0.04	0.21	0.41	0.76	1.24	1.73	2.12	2.36	2.49	2.55	2.58	2.60	2.60	2.61	2.61	2.61	2.61
Italy	0.06	0.13	0.18	0.49	1.57	3.25	4.32	4.68	4.78	4.80	4.80	4.80	4.80	4.80	4.80	4.80	4.80	4.80	4.80
Japan	0.15	0.17	0.25	1.19	2.36	4.30	6.92	9.64	11.77	13.11	13.83	14.19	14.37	14.45	14.49	14.50	14.51	14.52	14.52
Korea	0.14	0.24	0.39	1.80	3.59	6.59	10.69	14.94	18.27	20.34	21.46	22.02	22.28	22.40	22.46	22.49	22.50	22.50	22.51
Mexico	0.00	0.01	0.01	0.05	0.10	0.19	0.31	0.43	0.53	0.59	0.62	0.64	0.65	0.65	0.65	0.65	0.65	0.65	0.65
Netherlands	0.19	0.26	0.36	0.97	3.02	6.19	8.29	9.03	9.23	9.28	9.30	9.30	9.30	9.30	9.30	9.30	9.30	9.30	9.30
New Zealand	0.02	0.03	0.05	0.17	0.77	1.76	2.21	2.30	2.32	2.32	2.32	2.32	2.32	2.32	2.32	2.32	2.32	2.32	2.32
Norway	0.35	0.46	0.61	1.72	5.14	10.41	14.07	15.46	15.85	15.96	15.99	15.99	15.99	15.99	15.99	15.99	15.99	15.99	15.99
Other Europe	0.05	0.09	0.17	0.45	1.45	3.00	3.98	4.31	4.39	4.41	4.41	4.41	4.42	4.42	4.42	4.42	4.42	4.42	4.42
Poland	0.01	0.02	0.03	0.09	0.30	0.62	0.82	0.89	0.91	0.91	0.91	0.91	0.91	0.91	0.91	0.91	0.91	0.91	0.91
Portugal	0.02	0.04	0.06	0.16	0.49	1.01	1.34	1.46	1.49	1.50	1.50	1.50	1.50	1.50	1.50	1.50	1.50	1.50	1.50
Rest of the World	0.02	0.03	0.06	0.25	0.46	0.81	1.35	2.09	2.93	3.74	4.37	4.80	5.07	5.22	5.30	5.35	5.37	5.39	5.39

South Africa	0.00	0.00	0.00	0.01	0.01	0.02	0.04	0.05	0.07	0.08	0.09	0.10	0.10	0.10	0.10	0.10	0.10	0.10	0.10
Spain	0.05	0.08	0.11	0.30	0.94	1.92	2.58	2.81	2.87	2.89	2.89	2.90	2.90	2.90	2.90	2.90	2.90	2.90	2.90
Sweden	0.06	0.12	0.21	0.56	1.84	3.82	5.06	5.46	5.56	5.58	5.59	5.59	5.59	5.59	5.59	5.59	5.59	5.59	5.59
Switzerland	0.06	0.09	0.14	0.37	1.17	2.40	3.20	3.48	3.56	3.58	3.58	3.58	3.58	3.58	3.58	3.58	3.58	3.58	3.58
Turkey	0.00	0.01	0.01	0.07	0.13	0.24	0.39	0.54	0.66	0.74	0.78	0.80	0.81	0.81	0.81	0.82	0.82	0.82	0.82
USA	1.10	1.50	2.10	14.99	71.56	165.09	205.37	213.18	214.40	214.59	214.62	214.62	214.62	214.62	214.62	214.62	214.62	214.62	214.62
United Kingdom	0.21	0.41	0.59	1.58	5.05	10.43	13.89	15.06	15.37	15.44	15.46	15.46	15.46	15.46	15.46	15.46	15.46	15.46	15.46
Total	7.94	12.73	20.38	64.20	185.39	381.64	523.30	613.35	673.70	710.43	730.52	740.77	745.81	748.24	749.40	749.95	750.22	750.34	750.40

Table S2 | BEV stock across 33 major countries and regions (includes the "Rest of the World") under APS from 2010 to 2100 (units: million vehicles).

Name	2020	2021	2022	2025	2030	2035	2040	2045	2050	2055	2060	2065	2070	2075	2080	2085	2090	2095	2100
Australia	0.02	0.03	0.07	0.26	1.39	3.30	4.03	4.15	4.17	4.17	4.17	4.17	4.17	4.17	4.17	4.17	4.17	4.17	4.17
Austria	0.05	0.08	0.12	0.33	1.05	2.17	2.89	3.14	3.20	3.21	3.22	3.22	3.22	3.22	3.22	3.22	3.22	3.22	3.22
Belgium	0.03	0.05	0.09	0.25	0.84	1.76	2.33	2.51	2.56	2.57	2.57	2.57	2.57	2.57	2.57	2.57	2.57	2.57	2.57
Brazil	0.00	0.00	0.01	0.08	0.16	0.30	0.50	0.70	0.85	0.95	1.01	1.03	1.04	1.05	1.05	1.05	1.05	1.05	1.05
Canada	0.11	0.16	0.26	1.02	5.44	12.90	15.80	16.29	16.36	16.37	16.37	16.37	16.37	16.37	16.37	16.37	16.37	16.37	16.37
Chile	0.00	0.00	0.00	0.01	0.02	0.04	0.06	0.08	0.10	0.11	0.12	0.12	0.13	0.13	0.13	0.13	0.13	0.13	0.13
China	4.46	7.30	12.42	29.50	56.62	100.03	156.76	214.04	258.66	286.88	302.45	310.41	314.31	316.18	317.08	317.50	317.70	317.80	317.84
Denmark	0.03	0.07	0.12	0.31	1.03	2.16	2.85	3.07	3.13	3.14	3.14	3.14	3.14	3.14	3.14	3.14	3.14	3.14	3.14
Finland	0.01	0.02	0.05	0.12	0.42	0.89	1.18	1.26	1.28	1.29	1.29	1.29	1.29	1.29	1.29	1.29	1.29	1.29	1.29
France	0.34	0.51	0.70	1.93	6.14	12.68	16.98	18.46	18.86	18.95	18.97	18.98	18.98	18.98	18.98	18.98	18.98	18.98	18.98
Germany	0.36	0.73	1.06	2.87	9.43	19.65	26.05	28.14	28.66	28.78	28.80	28.81	28.81	28.81	28.81	28.81	28.81	28.81	28.81
Greece	0.00	0.00	0.01	0.02	0.07	0.15	0.19	0.21	0.21	0.21	0.21	0.21	0.21	0.21	0.21	0.21	0.21	0.21	0.21
Iceland	0.01	0.01	0.02	0.05	0.17	0.35	0.46	0.50	0.50	0.51	0.51	0.51	0.51	0.51	0.51	0.51	0.51	0.51	0.51
India	0.02	0.03	0.08	1.86	3.82	7.23	12.06	17.19	21.22	23.71	25.02	25.66	25.95	26.08	26.14	26.17	26.18	26.19	26.19
Israel	0.00	0.02	0.04	0.26	0.52	0.96	1.57	2.21	2.72	3.03	3.19	3.28	3.31	3.33	3.34	3.34	3.34	3.35	3.35
Italy	0.06	0.13	0.18	0.50	1.64	3.43	4.54	4.90	4.99	5.01	5.02	5.02	5.02	5.02	5.02	5.02	5.02	5.02	5.02
Japan	0.15	0.17	0.25	1.48	2.95	5.42	8.80	12.32	15.08	16.81	17.75	18.21	18.43	18.53	18.58	18.60	18.61	18.62	18.62
Korea	0.14	0.24	0.39	2.23	4.49	8.32	13.59	19.10	23.42	26.11	27.54	28.25	28.58	28.73	28.81	28.84	28.85	28.86	28.86
Mexico	0.00	0.01	0.01	0.06	0.13	0.24	0.39	0.55	0.68	0.76	0.80	0.82	0.83	0.83	0.84	0.84	0.84	0.84	0.84
Netherlands	0.19	0.26	0.36	0.99	3.16	6.54	8.72	9.46	9.65	9.70	9.71	9.71	9.71	9.71	9.71	9.71	9.71	9.71	9.71
New Zealand	0.02	0.03	0.05	0.19	0.99	2.35	2.88	2.96	2.98	2.98	2.98	2.98	2.98	2.98	2.98	2.98	2.98	2.98	2.98
Norway	0.35	0.46	0.61	1.74	5.40	11.06	14.86	16.21	16.58	16.68	16.70	16.70	16.71	16.71	16.71	16.71	16.71	16.71	16.71
Other Europe	0.05	0.09	0.17	0.45	1.51	3.17	4.19	4.51	4.59	4.61	4.61	4.61	4.61	4.61	4.61	4.61	4.61	4.61	4.61
Poland	0.01	0.02	0.03	0.09	0.31	0.65	0.86	0.93	0.95	0.95	0.95	0.95	0.95	0.95	0.95	0.95	0.95	0.95	0.95
Portugal	0.02	0.04	0.06	0.16	0.51	1.06	1.41	1.53	1.56	1.57	1.57	1.57	1.57	1.57	1.57	1.57	1.57	1.57	1.57
Rest of the World	0.02	0.03	0.06	0.32	0.58	1.02	1.71	2.66	3.76	4.80	5.62	6.17	6.51	6.70	6.81	6.86	6.89	6.91	6.92
South Africa	0.00	0.00	0.00	0.01	0.02	0.03	0.05	0.07	0.09	0.11	0.12	0.12	0.13	0.13	0.13	0.13	0.13	0.13	0.13
Spain	0.05	0.08	0.11	0.31	0.98	2.03	2.71	2.95	3.01	3.02	3.02	3.02	3.02	3.02	3.02	3.02	3.02	3.02	3.02
Sweden	0.06	0.12	0.21	0.57	1.92	4.02	5.31	5.71	5.81	5.83	5.84	5.84	5.84	5.84	5.84	5.84	5.84	5.84	5.84

Switzerland	0.06	0.09	0.14	0.38	1.22	2.54	3.37	3.65	3.72	3.74	3.74	3.74	3.74	3.74	3.74	3.74	3.74	3.74	3.74
Turkey	0.00	0.01	0.01	0.08	0.16	0.30	0.49	0.69	0.85	0.95	1.00	1.02	1.04	1.04	1.04	1.05	1.05	1.05	1.05
USA	1.10	1.50	2.10	15.12	70.35	161.43	201.78	209.89	211.20	211.40	211.43	211.44	211.44	211.44	211.44	211.44	211.44	211.44	211.44
United Kingdom	0.21	0.41	0.59	1.61	5.29	11.01	14.60	15.77	16.06	16.13	16.15	16.15	16.15	16.15	16.15	16.15	16.15	16.15	16.15
Total	7.94	12.73	20.38	65.16	188.75	389.18	533.98	625.83	687.46	725.02	745.59	756.10	761.27	763.77	764.96	765.53	765.80	765.93	765.99

Table S3 | PHEV stock across 33 major countries and regions (includes the "Rest of the World") under STEPS from 2010 to 2100 (units: million vehicles).

Name	2020	2021	2022	2025	2030	2035	2040	2045	2050	2055	2060	2065	2070	2075	2080	2085	2090	2095	2100
Australia	0.01	0.01	0.02	0.05	0.16	0.33	0.44	0.49	0.50	0.50	0.50	0.50	0.50	0.50	0.50	0.50	0.50	0.50	0.50
Austria	0.02	0.03	0.04	0.09	0.23	0.43	0.60	0.67	0.70	0.71	0.71	0.71	0.71	0.71	0.71	0.71	0.71	0.71	0.71
Belgium	0.07	0.12	0.18	0.38	0.97	1.84	2.55	2.89	3.01	3.05	3.06	3.07	3.07	3.07	3.07	3.07	3.07	3.07	3.07
Brazil	0.00	0.02	0.03	0.07	0.13	0.23	0.36	0.50	0.61	0.67	0.71	0.73	0.74	0.74	0.74	0.75	0.75	0.75	0.75
Canada	0.07	0.10	0.14	0.35	1.05	2.12	2.88	3.17	3.25	3.27	3.28	3.28	3.28	3.28	3.28	3.28	3.28	3.28	3.28
Chile	0.00	0.00	0.00	0.00	0.01	0.01	0.02	0.02	0.03	0.03	0.03	0.03	0.03	0.03	0.03	0.03	0.03	0.03	0.03
China	1.09	1.70	3.22	6.44	12.15	21.09	32.55	43.99	52.87	58.53	61.69	63.32	64.13	64.53	64.72	64.81	64.86	64.88	64.89
Denmark	0.03	0.08	0.10	0.21	0.55	1.06	1.45	1.63	1.70	1.71	1.72	1.72	1.72	1.72	1.72	1.72	1.72	1.72	1.72
Finland	0.05	0.08	0.10	0.21	0.54	1.02	1.41	1.60	1.67	1.70	1.70	1.71	1.71	1.71	1.71	1.71	1.71	1.71	1.71
France	0.13	0.27	0.37	0.78	2.00	3.82	5.27	5.95	6.20	6.28	6.30	6.31	6.31	6.31	6.31	6.31	6.31	6.31	6.31
Germany	0.30	0.63	0.89	1.86	4.84	9.26	12.74	14.35	14.91	15.08	15.14	15.15	15.16	15.16	15.16	15.16	15.16	15.16	15.16
Greece	0.00	0.01	0.01	0.02	0.07	0.13	0.17	0.20	0.20	0.20	0.20	0.20	0.20	0.20	0.20	0.20	0.20	0.20	0.20
Iceland	0.01	0.02	0.02	0.05	0.11	0.21	0.29	0.33	0.35	0.35	0.36	0.36	0.36	0.36	0.36	0.36	0.36	0.36	0.36
India	0.00	0.00	0.00	0.02	0.05	0.09	0.15	0.22	0.27	0.30	0.31	0.32	0.33	0.33	0.33	0.33	0.33	0.33	0.33
Israel	0.02	0.03	0.04	0.11	0.22	0.39	0.61	0.85	1.03	1.14	1.20	1.23	1.25	1.26	1.26	1.26	1.26	1.26	1.26
Italy	0.04	0.11	0.18	0.37	0.99	1.92	2.63	2.94	3.04	3.07	3.08	3.08	3.08	3.08	3.08	3.08	3.08	3.08	3.08
Japan	0.16	0.18	0.20	0.55	1.04	1.81	2.82	3.84	4.64	5.16	5.45	5.59	5.67	5.71	5.72	5.73	5.74	5.74	5.74
Korea	0.02	0.04	0.06	0.15	0.28	0.50	0.80	1.10	1.33	1.48	1.56	1.60	1.62	1.63	1.63	1.63	1.63	1.64	1.64
Mexico	0.01	0.01	0.01	0.04	0.07	0.12	0.20	0.27	0.33	0.36	0.38	0.39	0.40	0.40	0.40	0.40	0.40	0.40	0.40
Netherlands	0.11	0.14	0.19	0.43	0.95	1.70	2.42	2.88	3.09	3.18	3.22	3.23	3.23	3.24	3.24	3.24	3.24	3.24	3.24
New Zealand	0.01	0.01	0.02	0.05	0.15	0.31	0.41	0.45	0.45	0.46	0.46	0.46	0.46	0.46	0.46	0.46	0.46	0.46	0.46
Norway	0.15	0.18	0.20	0.45	1.03	1.87	2.64	3.09	3.29	3.37	3.40	3.41	3.41	3.41	3.41	3.41	3.41	3.41	3.41
Other Europe	0.03	0.05	0.09	0.18	0.48	0.92	1.26	1.41	1.46	1.48	1.49	1.49	1.49	1.49	1.49	1.49	1.49	1.49	1.49
Poland	0.01	0.02	0.03	0.06	0.16	0.31	0.43	0.49	0.50	0.51	0.51	0.51	0.51	0.51	0.51	0.51	0.51	0.51	0.51
Portugal	0.03	0.04	0.06	0.12	0.31	0.58	0.80	0.91	0.95	0.96	0.97	0.97	0.97	0.97	0.97	0.97	0.97	0.97	0.97
Rest of the World	0.01	0.02	0.02	0.05	0.09	0.15	0.25	0.37	0.51	0.65	0.76	0.83	0.88	0.91	0.92	0.93	0.94	0.94	0.94
South Africa	0.00	0.00	0.00	0.00	0.00	0.01	0.01	0.02	0.02	0.02	0.03	0.03	0.03	0.03	0.03	0.03	0.03	0.03	0.03
Spain	0.04	0.09	0.13	0.27	0.71	1.36	1.87	2.10	2.18	2.21	2.21	2.22	2.22	2.22	2.22	2.22	2.22	2.22	2.22
Sweden	0.12	0.19	0.24	0.52	1.28	2.40	3.34	3.82	4.00	4.06	4.08	4.09	4.09	4.09	4.09	4.09	4.09	4.09	4.09

Switzerland	0.03	0.05	0.07	0.15	0.38	0.72	1.00	1.14	1.19	1.20	1.21	1.21	1.21	1.21	1.21	1.21	1.21	1.21	1.21
Turkey	0.00	0.00	0.00	0.01	0.01	0.02	0.03	0.04	0.05	0.06	0.06	0.06	0.07	0.07	0.07	0.07	0.07	0.07	0.07
USA	0.64	0.72	0.86	3.73	11.37	23.16	31.16	34.08	34.88	35.09	35.14	35.16	35.16	35.16	35.16	35.16	35.16	35.16	35.16
United Kingdom	0.22	0.33	0.40	0.89	2.10	3.89	5.45	6.31	6.66	6.79	6.83	6.85	6.85	6.86	6.86	6.86	6.86	6.86	6.86
Total	3.43	5.28	7.94	18.66	44.46	83.79	119.01	142.08	155.88	163.65	167.75	169.83	170.85	171.35	171.59	171.70	171.76	171.79	171.80

Table S4 | PHEV stock across 33 major countries and regions (includes the "Rest of the World") under APS from 2010 to 2100 (units: million vehicles).

Name	2020	2021	2022	2025	2030	2035	2040	2045	2050	2055	2060	2065	2070	2075	2080	2085	2090	2095	2100
Australia	0.01	0.01	0.02	0.07	0.38	0.91	1.10	1.14	1.14	1.14	1.14	1.14	1.14	1.14	1.14	1.14	1.14	1.14	1.14
Austria	0.02	0.03	0.04	0.09	0.23	0.43	0.60	0.67	0.70	0.71	0.71	0.71	0.72	0.72	0.72	0.72	0.72	0.72	0.72
Belgium	0.07	0.12	0.18	0.38	0.97	1.84	2.55	2.89	3.01	3.05	3.07	3.07	3.07	3.07	3.07	3.07	3.07	3.07	3.07
Brazil	0.00	0.02	0.03	0.13	0.26	0.49	0.79	1.12	1.37	1.53	1.62	1.66	1.68	1.69	1.69	1.69	1.69	1.69	1.69
Canada	0.07	0.10	0.14	0.45	2.47	5.92	7.20	7.41	7.44	7.44	7.44	7.44	7.44	7.44	7.44	7.44	7.44	7.44	7.44
Chile	0.00	0.00	0.00	0.01	0.01	0.02	0.04	0.05	0.06	0.07	0.07	0.08	0.08	0.08	0.08	0.08	0.08	0.08	0.08
China	1.09	1.70	3.22	6.26	11.71	20.17	30.93	41.63	49.96	55.29	58.28	59.84	60.62	61.01	61.19	61.29	61.33	61.35	61.36
Denmark	0.03	0.08	0.10	0.21	0.55	1.06	1.45	1.63	1.70	1.72	1.72	1.72	1.72	1.72	1.72	1.72	1.72	1.72	1.72
Finland	0.05	0.08	0.10	0.21	0.54	1.02	1.41	1.60	1.67	1.70	1.70	1.71	1.71	1.71	1.71	1.71	1.71	1.71	1.71
France	0.13	0.27	0.37	0.78	2.01	3.82	5.27	5.96	6.20	6.28	6.31	6.31	6.32	6.32	6.32	6.32	6.32	6.32	6.32
Germany	0.30	0.63	0.89	1.87	4.85	9.27	12.75	14.35	14.92	15.09	15.15	15.16	15.17	15.17	15.17	15.17	15.17	15.17	15.17
Greece	0.00	0.01	0.01	0.02	0.07	0.13	0.17	0.20	0.20	0.20	0.20	0.20	0.20	0.20	0.20	0.20	0.20	0.20	0.20
Iceland	0.01	0.02	0.02	0.05	0.11	0.21	0.29	0.33	0.35	0.35	0.36	0.36	0.36	0.36	0.36	0.36	0.36	0.36	0.36
India	0.00	0.00	0.00	0.05	0.10	0.19	0.31	0.44	0.55	0.61	0.64	0.66	0.67	0.67	0.67	0.67	0.67	0.67	0.67
Israel	0.02	0.03	0.04	0.22	0.44	0.82	1.35	1.89	2.32	2.59	2.74	2.81	2.84	2.85	2.86	2.86	2.87	2.87	2.87
Italy	0.04	0.11	0.18	0.37	0.99	1.92	2.62	2.94	3.04	3.07	3.08	3.08	3.08	3.08	3.08	3.08	3.08	3.08	3.08
Japan	0.16	0.18	0.20	1.04	2.07	3.79	6.15	8.61	10.55	11.76	12.41	12.74	12.90	12.97	13.00	13.02	13.02	13.03	13.03
Korea	0.02	0.04	0.06	0.29	0.57	1.07	1.74	2.45	3.01	3.36	3.54	3.63	3.68	3.70	3.71	3.71	3.71	3.71	3.71
Mexico	0.01	0.01	0.01	0.07	0.14	0.26	0.43	0.60	0.74	0.82	0.87	0.89	0.90	0.91	0.91	0.91	0.91	0.91	0.91
Netherlands	0.11	0.14	0.19	0.44	0.95	1.71	2.42	2.88	3.09	3.18	3.22	3.23	3.24	3.24	3.24	3.24	3.24	3.24	3.24
New Zealand	0.01	0.01	0.02	0.06	0.34	0.83	1.01	1.03	1.04	1.04	1.04	1.04	1.04	1.04	1.04	1.04	1.04	1.04	1.04
Norway	0.15	0.18	0.20	0.45	1.03	1.87	2.64	3.09	3.29	3.37	3.40	3.41	3.41	3.41	3.41	3.42	3.42	3.42	3.42
Other Europe	0.03	0.05	0.09	0.18	0.48	0.92	1.26	1.41	1.47	1.48	1.49	1.49	1.49	1.49	1.49	1.49	1.49	1.49	1.49
Poland	0.01	0.02	0.03	0.06	0.16	0.31	0.43	0.48	0.50	0.51	0.51	0.51	0.51	0.51	0.51	0.51	0.51	0.51	0.51
Portugal	0.03	0.04	0.06	0.12	0.31	0.58	0.80	0.91	0.95	0.97	0.97	0.97	0.97	0.97	0.97	0.97	0.97	0.97	0.97
Rest of the World	0.01	0.02	0.02	0.10	0.18	0.32	0.53	0.82	1.16	1.48	1.74	1.91	2.01	2.07	2.10	2.12	2.13	2.14	2.14
South Africa	0.00	0.00	0.00	0.00	0.01	0.01	0.02	0.03	0.05	0.05	0.06	0.06	0.06	0.07	0.07	0.07	0.07	0.07	0.07
Spain	0.04	0.09	0.13	0.27	0.71	1.36	1.87	2.10	2.18	2.21	2.22	2.22	2.22	2.22	2.22	2.22	2.22	2.22	2.22
Sweden	0.12	0.19	0.24	0.52	1.28	2.40	3.34	3.82	4.00	4.06	4.09	4.09	4.09	4.09	4.10	4.10	4.10	4.10	4.10

Switzerland	0.03	0.05	0.07	0.15	0.38	0.72	1.00	1.14	1.19	1.20	1.21	1.21	1.21	1.21	1.21	1.21	1.21	1.21	1.21
Turkey	0.00	0.00	0.00	0.01	0.02	0.04	0.07	0.10	0.12	0.14	0.14	0.15	0.15	0.15	0.15	0.15	0.15	0.15	0.15
USA	0.64	0.72	0.86	3.73	11.19	22.66	30.61	33.60	34.45	34.68	34.73	34.75	34.75	34.75	34.75	34.75	34.75	34.75	34.75
United Kingdom	0.22	0.33	0.40	0.89	2.11	3.90	5.46	6.32	6.67	6.79	6.84	6.85	6.86	6.86	6.86	6.86	6.86	6.86	6.86
Total	3.43	5.28	7.94	19.55	47.61	90.97	128.64	153.68	169.11	177.96	182.70	185.11	186.30	186.88	187.17	187.30	187.37	187.40	187.42

Table S5 | The summary for estimated BEV and PHEV stock under STEPS and APS from 2010 to 2100 (units: million vehicles).

Scenario	Type	2020	2021	2022	2025	2030	2035	2040	2045	2050	2055	2060	2065	2070	2075	2080	2085	2090	2095	2100
**STEPS**	**BEV**	7.94	12.73	20.38	64.20	185.39	381.64	523.30	613.35	673.70	710.43	730.52	740.77	745.81	748.24	749.40	749.95	750.22	750.34	750.40
	**PHEV**	3.43	5.28	7.94	18.66	44.46	83.79	119.01	142.08	155.88	163.65	167.75	169.83	170.85	171.35	171.59	171.70	171.76	171.79	171.80
	**Total**	11.37	18.00	28.32	82.87	229.85	465.43	642.31	755.43	829.58	874.08	898.27	910.60	916.66	919.59	920.99	921.66	921.98	922.13	922.20
**APS**	**BEV**	7.94	12.73	20.38	65.16	188.75	389.18	533.98	625.83	687.46	725.02	745.59	756.10	761.27	763.77	764.96	765.53	765.80	765.93	765.99
	**PHEV**	3.43	5.28	7.94	19.55	47.61	90.97	128.64	153.68	169.11	177.96	182.70	185.11	186.30	186.88	187.17	187.30	187.37	187.40	187.42
	**Total**	11.37	18.00	28.32	84.71	236.36	480.15	662.62	779.51	856.56	902.99	928.29	941.21	947.58	950.65	952.13	952.83	953.17	953.33	953.41

Table S6 | The historical electricity demand for EVs under Ideal and Actual Scenario with different technical efficiency coefficients settings (TWh).

Scenario	Technical Efficiency Coefficients	Types	2010	2011	2012	2013	2014	2015	2016	2017	2018	2019	2020	2021	2022
**Historical Ideal Scenario**	**Baseline**	**BEV**	0.103	0.194	0.403	0.789	1.427	2.416	3.780	5.620	8.590	12.617	17.127	24.588	37.756
		**PHEV**	0.001	0.034	0.243	0.633	1.123	1.742	2.603	3.680	5.161	6.752	8.587	11.675	16.423
		**Total**	0.104	0.228	0.646	1.422	2.550	4.158	6.383	9.300	13.751	19.369	25.714	36.263	54.179
**Historical Actual Scenario**	**Baseline**	**BEV**	0.115	0.213	0.446	0.878	1.586	2.688	4.215	6.296	9.676	14.195	19.248	27.635	42.448
		**PHEV**	0.001	0.039	0.272	0.71	1.251	1.931	2.885	4.106	5.798	7.603	9.636	13.105	18.372
		**Total**	0.116	0.252	0.718	1.588	2.837	4.619	7.1	10.402	15.474	21.798	28.884	40.74	60.82
	**Upper Limit**	**BEV**	0.13	0.242	0.519	1.032	1.86	3.131	4.882	7.419	11.68	16.943	22.985	33.731	51.963
		**PHEV**	0.001	0.044	0.316	0.834	1.468	2.249	3.342	4.837	6.999	9.079	11.506	15.994	22.491
		**Total**	0.131	0.286	0.835	1.866	3.328	5.38	8.224	12.256	18.679	26.022	34.491	49.725	74.454
	**Lower Limit**	**BEV**	0.097	0.181	0.385	0.756	1.35	2.244	3.465	5.165	7.922	11.492	15.623	22.674	34.793
		**PHEV**	0.001	0.033	0.234	0.611	1.065	1.612	2.372	3.368	4.748	6.156	7.821	10.752	15.059
		**Total**	0.098	0.214	0.619	1.367	2.415	3.856	5.837	8.533	12.67	17.648	23.444	33.426	49.852

Table S7 | The ISO 3166-1 alpha-2 standard for main countries.

Name	ISO 3166-1 alpha-2 standard
South Africa	ZA
Chile	CL
Greece	GR
Mexico	MX
Brazil	BR
Turkey	TR
Australia	AU
Portugal	PT
Israel	IL
Poland	PL
Iceland	IS
Spain	ES
New Zealand	NZ
Belgium	BE
Italy	IT
Austria	AT
Denmark	DK
Rest of the World	ROW
Switzerland	CH
Finland	FI
Netherlands	NL
Japan	JP
France	FR
Korea	KR
India	IN
United Kingdom	GB
Sweden	SE
Other Europe	OE
Germany	DE
Norway	NO
Canada	CA
China	CN
USA	US

Table S8 | Electricity demand for EVs under different RCP-SSPs (TWh).

RCP-SSP GCM	2020	2021	2022	2025	2030	2035	2040	2045	2050	2055	2060	2065	2070	2075	2080	2085	2090	2095	2100
RCP-SSP126 CanESM5	29.3	41.1	61.5	220.1	684.2	1481.4	1990.5	2254.9	2465.2	2638.8	2773.3	2905.7	2996.5	3117.5	3203.5	3286.4	3407.5	3516.7	3590.1
RCP-SSP126 EC-Earth3	29.1	41.1	61.5	220.0	686.0	1491.1	1983.3	2241.8	2469.4	2657.7	2796.0	2928.5	3009.8	3103.2	3201.7	3321.7	3419.6	3516.0	3620.4
RCP-SSP126 FGOALS-g3	29.9	42.0	62.9	223.1	700.4	1524.8	2031.6	2310.6	2514.7	2710.0	2862.9	2996.3	3077.7	3184.7	3277.6	3377.3	3492.2	3587.9	3696.0
RCP-SSP126 GFDL-ESM4	29.3	41.3	61.8	219.7	688.1	1497.8	2012.2	2278.4	2480.6	2662.2	2823.3	2932.3	3047.5	3131.8	3225.8	3343.2	3442.4	3543.4	3630.3
RCP-SSP126 IPSL-CM6A-LR	29.8	41.8	62.5	222.3	693.3	1502.5	2018.9	2284.3	2483.3	2685.4	2828.6	2952.1	3035.9	3160.3	3246.0	3344.5	3450.4	3557.8	3635.8
RCP-SSP126 MIROC-ES2L	28.7	40.5	60.4	214.5	677.7	1465.4	1965.1	2224.7	2415.9	2594.6	2750.0	2890.7	2971.6	3072.5	3151.9	3252.7	3346.9	3454.0	3537.8
RCP-SSP126 MIROC6	28.6	40.5	60.6	215.0	669.5	1465.3	1974.0	2225.3	2426.4	2602.5	2758.9	2872.5	2974.9	3076.9	3170.3	3267.4	3377.4	3465.8	3554.2
RCP-SSP126 MPI-ESM1-2-LR	29.1	41.1	61.4	219.6	685.5	1486.2	2002.6	2256.8	2480.5	2664.2	2785.1	2929.8	3013.0	3141.0	3221.0	3316.7	3408.9	3505.0	3592.1
RCP-SSP126 MRI-ESM2-0	28.9	40.7	60.8	217.0	680.9	1472.3	1974.5	2245.9	2449.0	2608.1	2769.2	2877.1	2990.4	3094.2	3183.2	3294.5	3394.9	3497.4	3602.1
RCP-SSP126 NCAR_CESM2	28.7	40.4	60.4	214.9	671.4	1452.1	1948.1	2204.7	2411.6	2592.7	2752.5	2859.7	2960.0	3047.0	3145.5	3248.8	3343.7	3431.2	3526.3
RCP-SSP126 UKESM1-0-LL	29.4	41.4	61.9	221.6	695.2	1506.8	2020.4	2282.1	2470.4	2652.8	2797.6	2937.7	3044.4	3112.2	3203.7	3308.4	3415.7	3492.9	3579.7
RCP-SSP126 Ideal Scenario	25.8	36.4	54.5	195.1	611.0	1322.2	1780.0	2017.5	2201.3	2368.6	2498.5	2605.0	2699.1	2785.2	2871.6	2959.8	3045.7	3127.8	3206.5
RCP-SSP245 CanESM5	29.0	40.8	60.9	215.6	673.0	1425.1	1885.2	2111.7	2299.6	2460.2	2573.9	2692.4	2777.6	2867.1	2950.7	3022.6	3111.4	3179.0	3253.2
RCP-SSP245 EC-Earth3	29.2	40.8	61.1	215.3	673.7	1431.0	1910.9	2143.9	2294.2	2462.7	2593.1	2706.4	2816.6	2881.1	2977.2	3060.3	3138.8	3208.9	3273.6
RCP-SSP245 FGOALS-g3	29.8	42.0	62.4	221.2	683.1	1451.6	1930.1	2155.6	2354.3	2529.0	2649.5	2762.3	2866.9	2938.8	3034.3	3124.1	3207.1	3270.0	3369.3
RCP-SSP245 GFDL-ESM4	29.1	41.0	60.9	217.4	673.4	1434.6	1906.4	2144.1	2330.8	2491.9	2614.8	2730.5	2830.5	2898.0	2983.8	3069.1	3154.9	3226.2	3309.7
RCP-SSP245 IPSL-CM6A-LR	29.4	41.4	61.9	218.7	679.5	1452.4	1895.9	2148.5	2322.7	2489.3	2625.2	2741.8	2813.2	2915.8	3009.2	3065.5	3147.0	3226.5	3315.8
RCP-SSP245 MIROC-ES2L	28.5	40.0	59.6	213.5	662.7	1409.4	1868.0	2098.5	2267.9	2427.0	2549.7	2657.8	2757.3	2857.6	2912.6	3011.6	3083.1	3155.9	3239.1
RCP-SSP245 MIROC6	28.6	40.4	60.1	213.1	661.5	1412.2	1876.9	2101.3	2292.1	2429.7	2550.5	2662.2	2768.1	2868.0	2947.3	3032.8	3112.5	3189.1	3236.9
RCP-SSP245 MPI-ESM1-2-LR	29.2	40.7	60.4	216.2	671.0	1436.2	1910.7	2126.6	2294.3	2459.2	2590.4	2703.3	2802.0	2892.9	2973.2	3061.8	3120.0	3218.9	3289.3
RCP-SSP245 MRI-ESM2-0	28.8	40.3	60.2	214.0	662.0	1410.8	1878.1	2098.1	2271.6	2449.1	2573.1	2681.6	2788.2	2869.6	2951.8	3025.4	3112.2	3207.9	3281.3
RCP-SSP245 NCAR_CESM2	28.5	40.2	59.8	212.2	656.4	1408.8	1869.0	2082.5	2267.5	2423.9	2558.9	2665.6	2746.8	2840.1	2917.1	3005.4	3094.2	3163.9	3230.5
RCP-SSP245 UKESM1-0-LL	29.3	41.1	61.2	218.3	676.2	1463.2	1923.3	2151.0	2315.3	2487.5	2613.5	2720.4	2798.5	2875.9	2969.1	3054.7	3137.0	3187.5	3275.8
RCP-SSP245 Ideal Scenario	25.6	36.1	53.9	192.2	596.1	1273.6	1694.1	1902.5	2063.3	2212.6	2330.2	2431.1	2518.1	2598.5	2677.0	2753.2	2827.6	2897.1	2968.7
RCP-SSP370 CanESM5	29.1	40.6	60.6	214.2	660.4	1405.0	1858.0	2033.5	2176.0	2297.4	2380.6	2445.2	2494.1	2522.5	2563.2	2602.4	2638.5	2676.4	2698.8
RCP-SSP370 EC-Earth3	28.9	40.5	60.5	215.1	667.6	1415.5	1852.5	2055.9	2212.3	2326.3	2405.3	2490.6	2523.2	2565.0	2609.6	2632.7	2660.9	2701.4	2732.9
RCP-SSP370 FGOALS-g3	29.7	41.7	62.2	217.3	675.2	1431.5	1877.4	2086.7	2223.2	2369.5	2447.0	2532.3	2573.7	2625.4	2650.5	2694.0	2726.7	2755.9	2794.9

RCP-SSP370 GFDL-ESM4	29.2	40.8	60.9	215.5	670.6	1424.2	1882.9	2076.8	2221.9	2336.5	2418.5	2502.0	2554.3	2583.3	2618.4	2662.9	2699.1	2738.4	2777.8
RCP-SSP370 IPSL-CM6A-LR	29.6	41.3	61.6	217.3	673.7	1419.2	1874.8	2079.9	2214.4	2334.6	2450.4	2500.3	2559.7	2584.1	2623.2	2642.4	2684.0	2718.0	2759.1
RCP-SSP370 MIROC-ES2L	28.5	39.9	59.7	210.6	658.7	1386.2	1834.9	2021.5	2161.8	2278.7	2368.8	2453.2	2487.3	2517.5	2561.1	2597.2	2634.0	2677.0	2717.5
RCP-SSP370 MIROC6	28.5	40.0	59.5	211.7	655.3	1389.0	1827.5	2021.9	2171.6	2272.3	2372.2	2432.8	2488.5	2538.1	2557.3	2609.9	2645.7	2672.7	2715.3
RCP-SSP370 MPI-ESM1-2-LR	29.0	40.7	61.0	213.8	667.6	1429.1	1870.4	2056.0	2205.6	2332.5	2414.5	2484.0	2533.6	2570.7	2604.9	2645.7	2676.7	2718.5	2759.9
RCP-SSP370 MRI-ESM2-0	28.7	40.2	59.8	211.8	653.6	1403.9	1830.5	2041.1	2170.8	2309.9	2383.4	2457.1	2509.9	2548.2	2592.2	2624.9	2664.1	2692.0	2747.4
RCP-SSP370 NCAR_CESM2	28.5	39.9	59.4	211.2	654.7	1391.1	1836.2	2026.5	2150.8	2288.0	2363.9	2444.2	2489.0	2539.6	2573.0	2594.1	2645.5	2682.8	2715.7
RCP-SSP370 UKESM1-0-LL	29.4	40.9	60.9	215.5	670.3	1420.6	1873.6	2075.8	2203.0	2322.5	2406.6	2478.8	2516.2	2569.1	2600.7	2607.2	2652.4	2690.2	2713.6
RCP-SSP370 Ideal Scenario	25.6	36.0	53.7	190.5	589.4	1254.8	1656.5	1838.7	1968.5	2083.1	2166.1	2229.8	2280.1	2321.4	2360.5	2396.5	2433.0	2470.6	2510.6
RCP-SSP585 CanESM5	29.0	40.8	61.4	220.3	698.0	1520.2	2070.2	2374.1	2616.1	2855.3	3021.7	3202.1	3332.8	3479.8	3617.2	3746.1	3878.2	3998.2	4120.2
RCP-SSP585 EC-Earth3	29.1	41.0	61.2	219.6	694.8	1525.0	2080.0	2384.9	2637.5	2858.8	3060.0	3220.7	3364.1	3513.2	3642.6	3776.8	3915.6	4037.9	4153.8
RCP-SSP585 FGOALS-g3	29.7	41.8	62.2	223.6	713.3	1564.1	2139.0	2422.0	2672.5	2904.5	3115.3	3299.9	3454.5	3583.6	3738.2	3854.3	4006.1	4122.5	4239.0
RCP-SSP585 GFDL-ESM4	29.3	41.1	61.1	221.5	702.8	1546.8	2101.6	2409.2	2650.8	2870.9	3093.7	3248.2	3408.2	3550.3	3691.9	3824.1	3955.9	4089.5	4203.5
RCP-SSP585 IPSL-CM6A-LR	29.9	41.7	62.5	222.4	701.0	1538.0	2114.7	2406.7	2652.6	2890.5	3081.7	3247.7	3391.9	3534.2	3655.7	3806.6	3924.6	4051.7	4153.6
RCP-SSP585 MIROC-ES2L	28.6	40.4	60.5	216.3	684.3	1499.6	2049.9	2341.1	2595.9	2826.5	3020.4	3166.1	3322.2	3460.1	3594.1	3752.2	3878.6	3999.5	4109.4
RCP-SSP585 MIROC6	28.5	40.2	60.0	216.1	685.9	1511.2	2053.8	2349.3	2584.2	2821.7	3028.4	3202.7	3335.6	3466.9	3605.8	3752.8	3884.2	4013.2	4121.4
RCP-SSP585 MPI-ESM1-2-LR	29.0	40.6	60.8	220.0	702.3	1548.1	2103.3	2392.6	2640.9	2870.9	3061.9	3248.6	3362.7	3518.5	3675.4	3785.4	3944.0	4069.0	4183.4
RCP-SSP585 MRI-ESM2-0	28.7	40.4	60.1	216.8	691.7	1517.2	2052.8	2362.4	2610.2	2836.2	3031.6	3200.2	3355.5	3487.2	3633.7	3774.3	3915.3	4043.7	4161.5
RCP-SSP585 NCAR_CESM2	28.5	39.9	59.8	214.6	682.7	1502.6	2036.9	2355.4	2595.6	2818.9	2999.1	3171.2	3320.0	3455.6	3591.3	3737.7	3864.6	3995.3	4115.1
RCP-SSP585 UKESM1-0-LL	29.2	41.1	61.7	223.3	706.4	1546.1	2103.8	2403.0	2637.3	2865.0	3041.1	3216.4	3368.2	3505.1	3634.2	3778.9	3901.8	4022.5	4138.0
RCP-SSP585 Ideal Scenario	25.7	36.2	54.2	195.5	621.6	1366.5	1865.9	2141.7	2368.8	2584.4	2765.3	2925.1	3069.9	3205.8	3339.9	3473.6	3604.3	3727.7	3843.7
Averaged Actual Electricity Demand	29.1	40.8	60.9	217.1	679.0	1464.0	1956.8	2203.4	2395.1	2569.7	2708.1	2828.4	2923.5	3015.1	3102.1	3190.9	3279.9	3362.9	3442.5

Table S9 | The ratio of actual electricity demand for EVs to the ideal demand under different RCP-SSPs

RCP-SSP GCM	2020	2021	2022	2025	2030	2035	2040	2045	2050	2055	2060	2065	2070	2075	2080	2085	2090	2095	2100
RCP-SSP126 CanESM5	1.1357	1.1299	1.1286	1.1283	1.1198	1.1204	1.1183	1.1177	1.1199	1.1141	1.1100	1.1154	1.1102	1.1193	1.1156	1.1103	1.1188	1.1243	1.1196
RCP-SSP126 EC-Earth3	1.1270	1.1288	1.1284	1.1276	1.1227	1.1278	1.1142	1.1112	1.1218	1.1221	1.1191	1.1242	1.1151	1.1142	1.1150	1.1222	1.1228	1.1241	1.1291
RCP-SSP126 FGOALS-g3	1.1585	1.1541	1.1539	1.1438	1.1463	1.1532	1.1413	1.1453	1.1424	1.1441	1.1458	1.1502	1.1403	1.1434	1.1414	1.1410	1.1466	1.1471	1.1527
RCP-SSP126 GFDL-ESM4	1.1359	1.1337	1.1350	1.1264	1.1261	1.1328	1.1304	1.1293	1.1269	1.1240	1.1300	1.1257	1.1291	1.1245	1.1234	1.1295	1.1302	1.1329	1.1322
RCP-SSP126 IPSL-CM6A-LR	1.1562	1.1469	1.1474	1.1394	1.1346	1.1364	1.1342	1.1323	1.1281	1.1337	1.1321	1.1333	1.1248	1.1347	1.1304	1.1300	1.1329	1.1375	1.1339
RCP-SSP126 MIROC-ES2L	1.1138	1.1130	1.1091	1.0994	1.1092	1.1083	1.1040	1.1027	1.0975	1.0954	1.1007	1.1097	1.1009	1.1031	1.0976	1.0989	1.0989	1.1043	1.1033
RCP-SSP126 MIROC6	1.1080	1.1110	1.1117	1.1019	1.0957	1.1083	1.1090	1.1030	1.1023	1.0988	1.1042	1.1027	1.1022	1.1047	1.1040	1.1039	1.1089	1.1081	1.1084
RCP-SSP126 MPI-ESM1-2-LR	1.1284	1.1276	1.1273	1.1258	1.1219	1.1240	1.1250	1.1186	1.1269	1.1248	1.1147	1.1247	1.1163	1.1278	1.1217	1.1205	1.1192	1.1206	1.1203
RCP-SSP126 MRI-ESM2-0	1.1191	1.1175	1.1155	1.1121	1.1144	1.1135	1.1093	1.1132	1.1126	1.1011	1.1084	1.1045	1.1079	1.1109	1.1085	1.1131	1.1147	1.1182	1.1234
RCP-SSP126 NCAR_CESM2	1.1115	1.1085	1.1078	1.1015	1.0988	1.0982	1.0944	1.0928	1.0955	1.0946	1.1017	1.0978	1.0966	1.0940	1.0954	1.0976	1.0978	1.0970	1.0997
RCP-SSP126 UKESM1-0-LL	1.1387	1.1363	1.1360	1.1358	1.1378	1.1396	1.1350	1.1312	1.1223	1.1200	1.1197	1.1277	1.1279	1.1174	1.1157	1.1177	1.1215	1.1168	1.1164
RCP-SSP126 Ideal Scenario	1.0000	1.0000	1.0000	1.0000	1.0000	1.0000	1.0000	1.0000	1.0000	1.0000	1.0000	1.0000	1.0000	1.0000	1.0000	1.0000	1.0000	1.0000	1.0000
RCP-SSP245 CanESM5	1.1324	1.1290	1.1283	1.1217	1.1290	1.1190	1.1128	1.1100	1.1146	1.1119	1.1046	1.1075	1.1030	1.1034	1.1022	1.0978	1.1004	1.0973	1.0958
RCP-SSP245 EC-Earth3	1.1367	1.1293	1.1335	1.1203	1.1302	1.1236	1.1280	1.1269	1.1119	1.1130	1.1128	1.1132	1.1185	1.1087	1.1121	1.1115	1.1100	1.1076	1.1027
RCP-SSP245 FGOALS-g3	1.1627	1.1625	1.1564	1.1508	1.1460	1.1398	1.1393	1.1330	1.1411	1.1430	1.1370	1.1362	1.1385	1.1310	1.1335	1.1347	1.1342	1.1287	1.1350
RCP-SSP245 GFDL-ESM4	1.1340	1.1348	1.1283	1.1312	1.1297	1.1264	1.1253	1.1270	1.1297	1.1262	1.1221	1.1231	1.1240	1.1153	1.1146	1.1147	1.1157	1.1136	1.1149
RCP-SSP245 IPSL-CM6A-LR	1.1481	1.1462	1.1483	1.1379	1.1399	1.1404	1.1191	1.1293	1.1257	1.1251	1.1266	1.1278	1.1172	1.1221	1.1241	1.1134	1.1129	1.1137	1.1169
RCP-SSP245 MIROC-ES2L	1.1095	1.1081	1.1052	1.1105	1.1118	1.1066	1.1026	1.1030	1.0992	1.0969	1.0942	1.0932	1.0950	1.0997	1.0880	1.0939	1.0904	1.0893	1.0911
RCP-SSP245 MIROC6	1.1143	1.1186	1.1136	1.1087	1.1098	1.1088	1.1079	1.1045	1.1109	1.0981	1.0945	1.0951	1.0992	1.1037	1.1010	1.1016	1.1007	1.1008	1.0903
RCP-SSP245 MPI-ESM1-2-LR	1.1402	1.1250	1.1203	1.1245	1.1257	1.1277	1.1278	1.1178	1.1120	1.1114	1.1117	1.1119	1.1127	1.1133	1.1106	1.1121	1.1034	1.1111	1.1080
RCP-SSP245 MRI-ESM2-0	1.1241	1.1141	1.1162	1.1134	1.1106	1.1077	1.1086	1.1028	1.1010	1.1069	1.1042	1.1030	1.1072	1.1043	1.1027	1.0988	1.1006	1.1073	1.1053
RCP-SSP245 NCAR_CESM2	1.1115	1.1125	1.1096	1.1037	1.1013	1.1061	1.1033	1.0946	1.0990	1.0955	1.0981	1.0965	1.0908	1.0930	1.0897	1.0916	1.0943	1.0921	1.0882
RCP-SSP245 UKESM1-0-LL	1.1419	1.1373	1.1349	1.1357	1.1344	1.1489	1.1353	1.1306	1.1222	1.1242	1.1216	1.1190	1.1113	1.1067	1.1091	1.1095	1.1094	1.1002	1.1034
RCP-SSP245 Ideal Scenario	1.0000	1.0000	1.0000	1.0000	1.0000	1.0000	1.0000	1.0000	1.0000	1.0000	1.0000	1.0000	1.0000	1.0000	1.0000	1.0000	1.0000	1.0000	1.0000
RCP-SSP370 CanESM5	1.1352	1.1276	1.1287	1.1246	1.1205	1.1197	1.1217	1.1059	1.1054	1.1029	1.0990	1.0966	1.0939	1.0867	1.0859	1.0859	1.0845	1.0833	1.0750
RCP-SSP370 EC-Earth3	1.1303	1.1254	1.1271	1.1293	1.1328	1.1280	1.1183	1.1181	1.1239	1.1168	1.1104	1.1169	1.1066	1.1050	1.1055	1.0986	1.0937	1.0934	1.0885
RCP-SSP370 FGOALS-g3	1.1591	1.1569	1.1585	1.1405	1.1457	1.1408	1.1334	1.1349	1.1294	1.1375	1.1297	1.1356	1.1288	1.1310	1.1229	1.1242	1.1207	1.1155	1.1132

RCP-SSP370 GFDL-ESM4	1.1429	1.1335	1.1342	1.1314	1.1378	1.1350	1.1367	1.1295	1.1288	1.1217	1.1165	1.1220	1.1203	1.1128	1.1093	1.1112	1.1094	1.1084	1.1064
RCP-SSP370 IPSL-CM6A-LR	1.1585	1.1464	1.1475	1.1406	1.1432	1.1310	1.1318	1.1312	1.1249	1.1208	1.1312	1.1213	1.1226	1.1132	1.1113	1.1026	1.1032	1.1001	1.0990
RCP-SSP370 MIROC-ES2L	1.1124	1.1075	1.1126	1.1056	1.1177	1.1047	1.1077	1.0994	1.0982	1.0939	1.0936	1.1002	1.0909	1.0845	1.0850	1.0838	1.0826	1.0836	1.0824
RCP-SSP370 MIROC6	1.1124	1.1117	1.1078	1.1111	1.1119	1.1069	1.1032	1.0997	1.1032	1.0908	1.0951	1.0910	1.0914	1.0934	1.0834	1.0891	1.0875	1.0818	1.0816
RCP-SSP370 MPI-ESM1-2-LR	1.1338	1.1285	1.1359	1.1221	1.1328	1.1389	1.1292	1.1182	1.1204	1.1197	1.1147	1.1140	1.1112	1.1074	1.1036	1.1040	1.1002	1.1003	1.0993
RCP-SSP370 MRI-ESM2-0	1.1205	1.1151	1.1140	1.1116	1.1090	1.1188	1.1050	1.1101	1.1028	1.1089	1.1003	1.1019	1.1008	1.0977	1.0982	1.0953	1.0950	1.0896	1.0943
RCP-SSP370 NCAR_CESM2	1.1126	1.1080	1.1075	1.1086	1.1108	1.1087	1.1085	1.1021	1.0926	1.0984	1.0913	1.0961	1.0916	1.0940	1.0900	1.0825	1.0873	1.0859	1.0817
RCP-SSP370 UKESM1-0-LL	1.1470	1.1365	1.1354	1.1313	1.1373	1.1321	1.1311	1.1290	1.1192	1.1149	1.1110	1.1116	1.1036	1.1067	1.1018	1.0879	1.0902	1.0889	1.0808
RCP-SSP370 Ideal Scenario	1.0000	1.0000	1.0000	1.0000	1.0000	1.0000	1.0000	1.0000	1.0000	1.0000	1.0000	1.0000	1.0000	1.0000	1.0000	1.0000	1.0000	1.0000	1.0000
RCP-SSP585 CanESM5	1.1312	1.1253	1.1319	1.1271	1.1229	1.1125	1.1095	1.1085	1.1044	1.1048	1.0927	1.0947	1.0856	1.0855	1.0830	1.0784	1.0760	1.0726	1.0720
RCP-SSP585 EC-Earth3	1.1316	1.1304	1.1299	1.1234	1.1178	1.1160	1.1148	1.1136	1.1134	1.1062	1.1066	1.1011	1.0958	1.0959	1.0906	1.0873	1.0864	1.0832	1.0807
RCP-SSP585 FGOALS-g3	1.1556	1.1531	1.1474	1.1439	1.1475	1.1446	1.1464	1.1308	1.1282	1.1238	1.1266	1.1281	1.1253	1.1178	1.1193	1.1096	1.1115	1.1059	1.1028
RCP-SSP585 GFDL-ESM4	1.1423	1.1329	1.1279	1.1332	1.1306	1.1320	1.1263	1.1249	1.1190	1.1108	1.1187	1.1105	1.1102	1.1074	1.1054	1.1009	1.0976	1.0970	1.0936
RCP-SSP585 IPSL-CM6A-LR	1.1629	1.1495	1.1523	1.1375	1.1278	1.1255	1.1334	1.1237	1.1198	1.1185	1.1144	1.1103	1.1049	1.1024	1.0945	1.0959	1.0889	1.0869	1.0806
RCP-SSP585 MIROC-ES2L	1.1124	1.1152	1.1157	1.1063	1.1008	1.0974	1.0986	1.0931	1.0958	1.0937	1.0923	1.0824	1.0822	1.0793	1.0761	1.0802	1.0761	1.0729	1.0691
RCP-SSP585 MIROC6	1.1119	1.1102	1.1068	1.1054	1.1034	1.1059	1.1007	1.0969	1.0909	1.0918	1.0951	1.0949	1.0865	1.0814	1.0796	1.0804	1.0777	1.0766	1.0723
RCP-SSP585 MPI-ESM1-2-LR	1.1308	1.1198	1.1211	1.1254	1.1299	1.1329	1.1273	1.1171	1.1148	1.1109	1.1072	1.1106	1.0954	1.0975	1.1005	1.0898	1.0943	1.0916	1.0884
RCP-SSP585 MRI-ESM2-0	1.1172	1.1148	1.1091	1.1088	1.1127	1.1103	1.1002	1.1030	1.1019	1.0974	1.0963	1.0941	1.0930	1.0878	1.0880	1.0866	1.0863	1.0848	1.0827
RCP-SSP585 NCAR_CESM2	1.1102	1.1006	1.1034	1.0980	1.0983	1.0996	1.0917	1.0997	1.0957	1.0907	1.0846	1.0841	1.0815	1.0779	1.0753	1.0760	1.0722	1.0718	1.0706
RCP-SSP585 UKESM1-0-LL	1.1376	1.1329	1.1373	1.1422	1.1364	1.1314	1.1275	1.1220	1.1133	1.1086	1.0997	1.0996	1.0972	1.0934	1.0881	1.0879	1.0825	1.0791	1.0766
RCP-SSP585 Ideal Scenario	1.0000	1.0000	1.0000	1.0000	1.0000	1.0000	1.0000	1.0000	1.0000	1.0000	1.0000	1.0000	1.0000	1.0000	1.0000	1.0000	1.0000	1.0000	1.0000
Averaged Ratio	1.1318	1.1274	1.1270	1.1229	1.1233	1.1225	1.1188	1.1156	1.1138	1.1116	1.1100	1.1105	1.1070	1.1058	1.1035	1.1023	1.1020	1.1010	1.0996

Table S10 | Definitions of EV policy scenarios. All net zero emissions pledges included in the APS have been incorporated into the IEA Climate Pledges Explorer.[9]

Name	Definitions	Objectives
**STEPS (Stated Policies Scenario)**	The STEPS is a scenario that reflects the current policy landscape based on a detailed, sector-by-sector, and country-by-country evaluation of the energy-related policies in place as of the end of August 2023, as well as those under development. The scenario also considers the currently planned manufacturing capacities for clean energy technologies.	The purpose of the STEPS is to establish a benchmark to evaluate the potential achievements and limitations of recent developments in energy and climate policy. The differences between the STEPS and the APS emphasize the "implementation gap" that needs to be closed for countries to reach their announced decarbonization targets.
**APS (Announced Pledges Scenario)**	The APS is a scenario that assumes all climate commitments made by governments and industries worldwide as of the end of August 2023, including NDCs, longer-term net zero targets, and goals for access to electricity and clean cooking, will be fully achieved within the stated timeframes.	The purpose of the APS is to illustrate how close current pledges bring the world to the target of limiting global warming to 1.5°C. The differences between the APS and the Net Zero Emissions by 2050 Scenario highlight the "ambition gap" that needs to be bridged to achieve the goals set forth in the Paris Agreement adopted in 2015. It also demonstrates the gap between current targets and achieving universal energy access.

Table S11 | List of ensemble General Circulation Models (GCMs). This table provides descriptions of various GCMs from CMIP6, detailing their associated institutions and countries, key focuses, and specific variant labels, including CanESM5, EC-Earth3, FGOALS-g3, GFDL-ESM4, IPSL-CM6A-LR, MIROC6, MIROC-ES2L, MPI-ESM1-2-LR, MRI-ESM2-0, UKESM1-0-LL, and NCAR CESM2.

Name	Description	Variant labels
**CanESM5**	CanESM5 (Canadian Earth System Model 5) was developed by the Canadian Centre for Climate Modelling and Analysis (CCCma). It serves as a global climate model for long-term studies of climate change and variability. It particularly emphasizes integrated simulations of atmospheric, oceanic interactions, and biogeochemical cycling.	r1i1p1f1
**EC-Earth3**	EC-Earth3, developed by the EC-Earth Consortium, is a collaborative project comprising research institutions from multiple European countries. This model focuses on high-resolution global climate simulations, providing detailed representations of atmospheric, oceanic, and terrestrial processes.	r1i1p1f1
**FGOALS-g3**	FGOALS-g3 (Flexible Global Ocean-Atmosphere-Land System model, version g3) was developed by the Chinese Academy of Sciences' Institute of Atmospheric Physics (CAS). This model emphasizes comprehensive simulations of ocean-atmosphere-land systems, particularly excelling in simulating climate phenomena such as the El Niño-Southern Oscillation (ENSO).	r1i1p1f1
**GFDL-ESM4**	GFDL-ESM4 (Geophysical Fluid Dynamics Laboratory Earth System Model 4) was developed by the Geophysical Fluid Dynamics Laboratory (GFDL), a part of the National Oceanic and Atmospheric Administration (NOAA) in the United States. It has a long history and outstanding performance in simulating the physical, chemical, and biological processes of the global climate system.	r1i1p1f1
**IPSL-CM6A-LR**	IPSL-CM6A-LR (Institute Pierre Simon Laplace Coupled Model 6A - Low Resolution) was developed by the IPSL climate modeling center in France. This model particularly focuses on the interactions among the atmosphere, ocean, and terrestrial ecosystems, emphasizing the integration of biogeochemical cycles.	r1i1p1f1
**MIROC6**	MIROC6, developed by the MIROC (Model for Interdisciplinary Research on Climate) team in Japan, is an advanced Earth system model utilized for studying climate dynamics, atmospheric chemistry, and ecosystem interactions. It holds significant importance in global climate change prediction and Earth system science research.	r1i1p1f1
**MIROC-ES2L**	MIROC-ES2L (Model for Interdisciplinary Research on Climate - Earth System 2 Low-resolution), developed by the MIROC team in Japan, is a specialized Earth system model focused on low-resolution simulations. This specialization enhances its efficiency in	r1i1p1f2

	conducting long-term climate simulations and historical climate reconstructions.	
**MPI-ESM1-2-LR**	MPI-ESM1-2-LR (Max Planck Institute Earth System Model 1-2 - Low Resolution) was developed by the Max Planck Institute for Meteorology (MPI-M) in Germany. This model aims to understand the dynamic processes of the climate system and the impact of human activities on climate.	r1i1p1f1
**MRI-ESM2-0**	MRI-ESM2-0 (Meteorological Research Institute Earth System Model 2.0) was developed by the Meteorological Research Institute in Japan. This model focuses on accurately simulating interactions between the atmosphere and ocean, as well as extreme climate events.	r1i1p1f1
**UKESM1-0-LL**	UKESM1-0-LL (UK Earth System Model 1.0 - Low Resolution) was jointly developed by the United Kingdom Met Office and multiple research partners. This model integrates processes of the atmosphere, ocean, cryosphere, and terrestrial biogeochemistry, with a particular emphasis on the impact of climate change on ecosystems.	r1i1p1f2
**NCAR CESM2**	NCAR CESM2 (Community Earth System Model 2) was developed by the National Center for Atmospheric Research (NCAR) in the United States. CESM2 is an advanced Earth system model used for studying climate change on scales ranging from seasons to millennia, including detailed simulations of atmospheric, oceanic, terrestrial, sea ice, and biogeochemical components.	r4i1p1f1

Table S12 | Definition of the different Shared Socioeconomic Pathways (SSPs).

Name	Definition
**SSP1 (Sustainability)**	This scenario envisions a future world that shifts gradually, but pervasively, toward a more sustainable path. It assumes low population growth, high education and health investments, and a rapid transition toward clean energy technologies. There is an emphasis on human well-being, reduced inequality, and protection of the environment. Economic growth is relatively low and is focused on environmentally-friendly industries.
**SSP2 (Middle of the Road)**	This is a continuation of historical trends, with some progress towards achieving development goals, reducing resource and energy intensity, and decreasing fossil fuel dependency. Population growth and income growth are moderate and uneven. Globalization and international cooperation continue but are not prioritized. Environmental systems experience degradation, although there is some improvement in resource efficiency.
**SSP3 (Regional Rivalry)**	This scenario assumes a resurgent nationalism, concerns about competitiveness and security, and regional conflicts. Countries focus on achieving energy and food security goals within their own regions at the expense of broader-based development. Population growth is low in industrialized countries and high in developing ones. Economic development is slow, consumption is material-intensive, and inequalities persist or worsen over time.
**SSP4 (Inequality)**	This pathway envisions a highly unequal world both within and across countries. There is increasing stratification between a well-educated, internationally connected society and a poorer, lower educated society with limited access to public services. Economic growth is moderate in industrialized countries and low in developing ones. Environmental policies focus on local issues around middle and high income areas.

**SSP5 (Fossil-fueled Development)**	This world places increasing faith in competitive markets, innovation and participatory societies to produce rapid technological progress and development of human capital as the path to sustainable development. Global markets are increasingly integrated. There are also strong investments in health, education, and institutions to enhance human and social capital. At the same time, the push for economic and social development is coupled with the exploitation of abundant fossil fuel resources and the adoption of resource and energy intensive lifestyles around the world.

Table S13 | Definition of the different Representative Concentration Pathways-Shared Socioeconomic Pathway (RCP-SSPs) scenarios. The Scenario Model Intercomparison Project (ScenarioMIP) design (modified from O'Neill *et al.*[10] and Tebaldi *et al.*[11]) is indicated by white and colored boxes, which depict achievable 2100 levels of forcings under the different SSPs.[10,11] The gray areas denote the intersection of SSPs and radiative forcing levels that were not achievable by any of the IAMs employed to produce these scenarios. The deep green area represents scenarios widely adopted in previous studies.

Climate 2100 Forcing Level (W/m^2)	SSP1 Sustainability	SSP2 Middle of the Road	SSP3 Regional Rivalry	SSP4 Inequality	SSP5 Fossil-fueled Development	RCPs
**8.5**					LTE	
**7.0**			ENS			
**6.0**						
**4.5**						
**3.4**					OS+LTE	
**2.6**	LTE					
**1.9**						

Tier 1 | Tier 2 | High Applications

ENS: Initial condition ensemble

OS: Overshoot

LTE: Long-term extension

References

[1] IEA. Energy Technology Perspectives 2017, IEA, Paris https://www.iea.org/reports/energy-technology-perspectives-2017, Licence: CC BY 4.0. (2017).

[2] IEA. Global EV Outlook 2018, IEA, Paris https://www.iea.org/reports/global-ev-outlook-2018, Licence: CC BY 4.0. (2018).

[3] IEA. Global EV Outlook 2019, IEA, Paris https://www.iea.org/reports/global-ev-outlook-2019, Licence: CC BY 4.0. (2019).

[4] IEA. Global EV Outlook 2020, IEA, Paris https://www.iea.org/reports/global-ev-outlook-2020, Licence: CC BY 4.0. (2020).

[5] EV Database. Energy consumption of full electric vehicles. [online] Available at: https://ev-database.org/cheatsheet/energy-consumption-electric-car [Accessed 23 Mar. 2024]. (2024).

[6] EPA. Fuel Economy Guide 2011-2017. (2017).

[7] Hall, D. & Lutsey, N. Effects of battery manufacturing on electric vehicle life-cycle greenhouse gas emissions. (2018).

[8] Hou, F. *et al*. Comprehensive analysis method of determining global long-term GHG mitigation potential of passenger battery electric vehicles. Journal of cleaner production 289, 125137 (2021).

[9] IEA. Climate Pledges Explorer. [online] Available at: https://www.iea.org/data-and-statistics/data-tools/climate-pledges-explorer [Accessed 7 Jun. 2025]. (2023).

[10] O'Neill, B. C. *et al.* Achievements and needs for the climate change scenario framework. Nature climate change 10, 1074-1084 (2020).

[11] Tebaldi, C. *et al.* Climate model projections from the scenario model intercomparison project (ScenarioMIP) of CMIP6. Earth System Dynamics Discussions 2020, 1-50 (2020).
